# Land-Use and Depth-Dependent Assembly of Soil Microbiomes Shapes Ecological Functions, Interaction Networks, and Phytopathogenic Communities Across Crop and Orchard Systems

**DOI:** 10.3390/antiox15081017

**Published:** 2026-08-14

**Authors:** Njomza Gashi, Péter Dávid, Maja Mikolás, Péter Fauszt, Ferenc Gál, Csaba Rácz, Krisztina Molnár, László Stündl, Judit Remenyik, Attila Csaba Dobos, Melinda Paholcsek

**Affiliations:** 1 Center for Complex Systems and Microbiome Innovations, Faculty of Agricultural and Food Sciences and Environmental Management, University of Debrecen, 4032 Debrecen, Hungary; david.peter@agr.unideb.hu (P.D.); mikolas.maja@agr.unideb.hu (M.M.); fauszt.peter@agr.unideb.hu (P.F.); gal.ferenc.gabor@agr.unideb.hu (F.G.); remenyik@agr.unideb.hu (J.R.); 2 Department of Food Technology with Biotechnology, Faculty of Agriculture and Veterinary, University of Prishtina, 10 000 Prishtina, Kosovo; 3 Centre for Precision Farming R&D Services, Faculty of Agricultural and Food Sciences and Environmental Management, University of Debrecen, 4032 Debrecen, Hungary; raczcs@agr.unideb.hu (C.R.); molnark@agr.unideb.hu (K.M.); dobosa@agr.unideb.hu (A.C.D.); 4 Institute of Food Technology, Faculty of Agricultural and Food Sciences and Environmental Management, University of Debrecen, 4032 Debrecen, Hungary; stundl@agr.unideb.hu

**Keywords:** soil microbiome, shotgun metagenomics, land-use, crops, orchards, soil depth, oxidative stress response, phytopathogenic species

## Abstract

Soil microorganisms are essential for nutrient cycling, plant productivity, and soil health, yet the relative importance of land-use and soil depth in shaping agricultural microbiomes remains poorly understood. This study investigated soil microbial communities across uncultivated land, alfalfa fields, crop systems (feed corn and sweet corn), and orchard systems (walnut and quince) in the Hajdúnánás region of Hungary using shotgun metagenomic sequencing and soil physicochemical analyses. Microbial alpha diversity showed limited variation among land-use systems but declined significantly with soil depth in both bacterial (Kruskal–Wallis, *p* = 0.00054) and fungal (*p* = 0.00051) communities. Beta diversity analyses identified soil depth as the primary driver of microbial community composition in both bacterial (*R*^2^ = 0.305, *p* = 0.001) and fungal (*R*^2^ = 0.277, *p* = 0.001) communities. In contrast, land-use significantly influenced only fungal community composition (*R*^2^ = 0.250, *p* = 0.005). Fungal alpha diversity showed significant negative relationships with soil pH and CaCO_3_, whereas bacterial diversity exhibited only weak correlations. Crop soils contained the highest numbers of unique bacterial and fungal taxa. Functional analyses revealed significant differences in nutrient cycling, plant-growth-related, decomposition, and environmental adaptation functions among land-use systems. In crop soils, topsoil communities were enriched in oxidative stress-related pathways involved in reactive oxygen species detoxification (ROS), redox homeostasis, and stress regulation, whereas subsoil communities showed a greater representation of antioxidant metabolite production functions. Co-occurrence network analyses indicated greater connectivity in perennial systems, particularly alfalfa soils. Analyses of bacterial and fungal species with reported phytopathogenic potential identified stable cores of phytopathogenic species across agricultural systems, with soil pH emerging as the strongest environmental factor associated with the abundance of phytopathogenic species. Overall, soil depth was the primary driver of bacterial and fungal community assembly, whereas land-use primarily shaped fungal community composition and influenced ecological functions, microbial interaction networks, and the distribution of phytopathogenic species.

## 1. Introduction

Maximizing agricultural production is a primary objective for farmers today. However, it is crucial to achieve this goal while prioritizing human health, which relies on the nutritional quality of food. Modern food production heavily relies on the extensive use of fertilizers, various tillage systems, and other treatments, all of which significantly impact soil health. Studies indicate that different forms of soil treatment have a direct impact on soil health and productivity, influencing changes in its microbial composition [1]. Soil microorganisms play a crucial role in ecosystems by cycling nutrients, stabilizing soil, and aiding plant growth. They enhance disease resistance, regulate soil pH, boost soil organic carbon levels, and compete effectively against pathogens [2,3].

Soil microbial composition is primarily influenced by crops that are planted. For instance, orchards show a significant difference in soil composition compared to croplands. According to a study by Cheng et al. [1], orchard-planted soils have higher microbial diversity and impact the preservation of soil structure, especially in its surface layers. However, the continuous cultivation of fruit trees as monocultures has been shown to cause replant syndrome issues. This disease, resulting from the repeated planting of the same crops in the same field, has led to reduced yields, hindered growth and development of plants, and affected their nutritional composition [4]. This occurs because the pathogenic members of the soil community become more robust, increasing their resistance to agrochemicals, and acting synergistically to cause such problems [5]. Monocultures decrease microbial diversity and increase pathogen resistance to various agrochemicals. This resistance can be transferred through gene transfer, directly impacting soil productivity, and leading to the development of resistant strains. Soils planted with monocultures or fruit trees have higher levels of antibiotic resistance due to the more favorable conditions for microorganisms in these undisturbed environments, facilitating their easier spread [6]. Cover crops such as alfalfa, buckwheat, and vetch are an environmentally friendly solution with desirable outcomes in supporting soil health. They promote the development of beneficial microorganisms, including those that solubilize phosphate, fix nitrogen, and others, thereby contributing to the enhancement of soil health and the promotion of microbiological growth [7]. Crop rotation has also demonstrated its significant impact on carbon sequestration and the formation of soil structure, which are essential for supporting plant growth and enhancing nutrient availability [8]. The practice of rotating different crops in agricultural soil disrupts the life cycles of pathogenic microorganisms by creating less favorable conditions for their growth and proliferation. This aids to break the cycle of pests, diseases, and pathogens that may build up when the same crop is grown repeatedly in the same soil. By introducing crop rotation, the need for agrochemical application is reduced, thereby supporting sustainable agriculture practices.

Soil microbial composition is influenced by soil properties as well. The topographic position of agricultural land directly impacts microbial composition through the nutrients provided to plants and the available water content. For instance, fungal levels are decreased in lower topographic positions than in higher topographic positions, as fungi adapt more easily to challenging environments with limited nutrient availability [9]. Due to less favorable temperatures, nutrient stress and variations in soil characteristics, the microbial community, especially bacteria, thrives more abundantly at lower topographic positions and demonstrates a higher capability for decomposing organic carbon [10]. Moreover, such differences are obvious in different soil layers, where deeper layers are related to more restricted living conditions for microbes. This impacts microbial diversity in higher layers of soil compared to deeper layers [11,12]. Such differences affect the physicochemical parameters of soil, shaping the adaptation of microorganisms in specific soils.

In this study, we investigated the soil microbiome across contrasting agricultural land-use systems in the Hajdúnánás region of Hungary, including crop fields, orchards, alfalfa, and uncultivated soils. Using shotgun metagenomic sequencing combined with soil physicochemical analyses, we aimed to assess how land-use, soil depth, and environmental conditions influence microbial diversity, community composition, ecological functions, microbial interactions, and the distribution of phytopathogenic species. Specifically, this study aimed to: (i) characterize the diversity and composition of bacterial and fungal communities across contrasting land-use systems and soil depths; (ii) evaluate the relationships between microbial communities and soil physicochemical properties; (iii) investigate the functional potential and interaction networks of soil microorganisms; and (iv) assess the distribution, prevalence, and environmental drivers of bacterial and fungal species with reported phytopathogenic potential. By integrating taxonomic, functional, network, and phytopathogenic species analyses, this study provides a comprehensive assessment of the ecological mechanisms governing soil microbiome assembly and functioning in agricultural ecosystems. Although vertical stratification of soil microbial communities has been reported previously, most studies have focused primarily on taxonomic composition or alpha diversity. In contrast, the present study integrates taxonomic diversity, ecological functions, co-occurrence networks, and communities of bacterial and fungal species with reported phytopathogenic potential using shotgun metagenomic sequencing across contrasting agricultural land-use systems and soil depths. This integrative approach provides new insights into how soil depth influences not only microbial community structure but also functional potential, ecological interactions, and the distribution of phytopathogenic species, thereby extending current knowledge beyond the description of vertical stratification alone. Moreover, these findings provide practical guidance for sustainable agricultural management by improving our understanding of how different plant production systems shape soil microbial communities and their ecological functions. This knowledge may support the selection of appropriate cropping systems and management practices that promote soil health, enhance crop productivity, and reduce reliance on chemical fertilizers and pesticides.

## 2. Materials and Methods

### 2.1. Sampling

**Figure 1:**
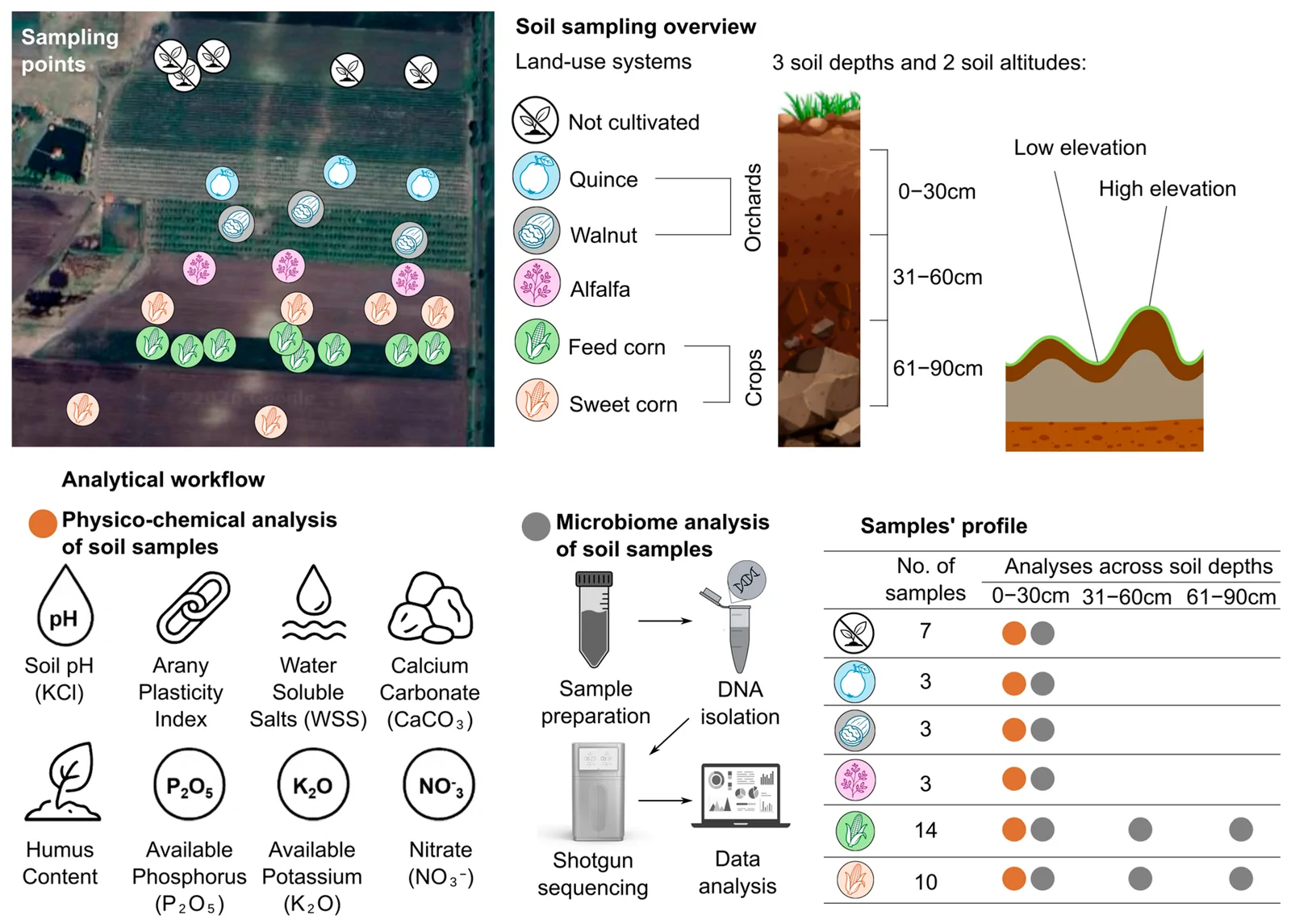
Schematic overview of the experimental design showing the land-use systems, soil sampling strategy, sampling depths, topographic positions, and analytical workflow.

The experiment was conducted in the Hajdúnánás region, Hungary (47°51′ N, 21°24′ E), including six agricultural management systems representing different land-use types. These included two crop production systems (feed corn and sweet corn), two orchard systems (quince and walnut), one alfalfa-covered area, and one unplanted control area. The control site was not cultivated with any crop during the study period and therefore served as a non-cropped reference site. Although the soil underwent occasional shallow loosening and ploughing for land maintenance, no planting, fertilization, irrigation, or crop management practices were applied. Consequently, this site represents a managed non-cropped agricultural reference site rather than an undisturbed natural ecosystem. A total of 28 sampling points were established across the six land-use systems (Figure 1). Soil sampling was carried out once during October and November, with the majority of the samples collected in November and the remaining five samples collected in October. Samples were collected from the 0–30, 31–60, and 61–90 cm soil depth intervals, resulting in a total of 40 soil samples. Sampling was performed at both higher and lower topographic positions. The higher and lower topographic sampling positions differed in elevation by approximately 1.36 m. The sampling scheme was designed to capture the main environmental gradients investigated in this study, namely land-use type, soil depth, and topographic position, thereby enabling comparisons of microbial diversity, community composition, ecological functions, and the distribution of species with reported phytopathogenic potential across contrasting agricultural systems. Sampling locations were selected according to the experimental land-use systems, soil depth, and topographic position to represent the main environmental gradients of the study rather than by random selection. A schematic overview of the sampling design, including the number of sampling points for each land-use type, soil depths, topographic positions, and analyses performed, is presented in Figure 1.

Following collection, soil samples were transferred into sterile Falcon tubes (SARSTEDT AG & Co. KG, Nümbrecht, Germany), transported under controlled conditions, and stored at −80 °C until further laboratory analyses.

Soil physicochemical analyses were performed according to the standard laboratory procedures described by Filep [13] at the University of Debrecen. Prior to analysis, soil samples were dried at 40 °C for 24 h, plant residues, roots, and stones were removed, and the samples were homogenized and passed through a 2 mm sieve. The investigated parameters included soil pH (measured in KCl), the Arany plasticity index (KA), water-soluble salts (WSS) (%m/m), calcium carbonate (CaCO_3_) (%m/m), humus content (%m/m), available phosphorus (P_2_O_5_) (mg/kg), available potassium (K_2_O) (mg/kg), and nitrate concentration (mg/kg). The Arany plasticity index (KA), a standard Hungarian soil physical parameter, was determined according to the Hungarian Standard MSZ-08-0206-2:1978. The KA index characterizes soil texture and plasticity, with higher values indicating finer-textured, more clay-rich soils. Water-soluble salts were determined as the total concentration of dissolved salts. Humus content was determined by wet oxidation of organic carbon using a 1:2 mixture of 5% potassium dichromate (K_2_Cr_2_O_7_) and concentrated sulfuric acid (H_2_SO_4_), and absorbance was measured using a Lambda 25 spectrophotometer (PerkinElmer Inc., Shelton, CT, USA). Available phosphorus (P_2_O_5_) and potassium (K_2_O) were determined using the ammonium lactate (AL) extraction method, whereas nitrate concentration was determined according to the procedures described by Filep [13].

### 2.2. DNA Extraction and Shotgun Sequencing

Microbial genomic DNA was extracted from soil samples using the DNeasy^®^ PowerSoil^®^ Pro Kit (QIAGEN GmbH, Hilden, Germany) following the manufacturer’s instructions with minor modifications [14]. Briefly, 5 g of fresh soil was suspended in 25 mL of sterile 1× phosphate-buffered saline (PBS) and vortexed for 3 min to detach microbial cells from soil particles. The suspension was centrifuged at 200× *g*, and the supernatant was transferred to a clean tube. This washing step was repeated twice, after which the collected supernatants were centrifuged at 12,000× *g* for 20 min to pellet microbial cells. The pellet was resuspended in 1 mL of sterile PBS, and 250 μL of the suspension was used for DNA extraction.

The microbial suspension was subsequently centrifuged at 21,000× *g* for 5 min, and the resulting pellet was resuspended in Solution CD1 and transferred into PowerBead Pro Tubes (QIAGEN GmbH, Hilden, Germany). Samples were incubated at 65 °C for 10 min and subjected to two cycles of mechanical lysis using a MagNA Lyser Instrument (Roche Applied Sciences, Mannheim, Germany) at 6000 rpm for 30 s each. DNA purification was then completed according to the manufacturer’s protocol, and purified DNA was eluted in 70 μL of Solution C6.

An indirect DNA extraction approach was employed, in which microbial cells were first concentrated from the soil suspension prior to lysis. This protocol was selected based on preliminary method optimization, which demonstrated that indirect extraction provided higher DNA yield and quality for whole-metagenome shotgun sequencing than direct DNA extraction from soil [15].

DNA concentration was quantified using a Qubit^®^ 4.0 Fluorometer (Thermo Fisher Scientific, Waltham, MA, USA), whereas DNA purity was assessed based on the OD260/280 ratio. Only DNA samples with an OD260/280 ratio between 1.8 and 2.0 and a concentration of at least 10 ng μL^−1^ were considered suitable for downstream whole-metagenome shotgun sequencing. Samples that did not meet these quality requirements were re-extracted until the required DNA quality and concentration were achieved. Sequencing libraries were prepared from purified metagenomic DNA through fragmentation and adapter ligation. Whole-metagenome shotgun sequencing was performed by Novogene Bioinformatics Technology (Beijing, China) on an Illumina NovaSeq 6000 platform (Illumina, San Diego, CA, USA), generating 150 bp paired-end reads. A sequencing depth of approximately 20 million paired-end reads per sample was achieved to ensure robust characterization of both taxonomic diversity and functional gene content.

### 2.3. Metagenomic Data Processing

Metagenomic data were processed using the SqueezeMeta pipeline (v1.6.3) with the co-assembly option and no binning. High-quality paired-end reads from all samples were co-assembled into contigs using MEGAHIT. These contigs were subsequently used for taxonomic classification with DIAMOND (v2.19) against the GenBank non-redundant (nr) database using the fast Lowest Common Ancestor (LCA) algorithm. This process generated the species-level taxonomic abundance matrix used for all downstream analyses. All bioinformatics analyses were carried out on the Komondor High-Performance Computing (HPC) cluster of the KIFÜ Hungarian High-Performance Computing Competence Center using 48 CPU cores and 90 GB RAM per sample. The resulting taxonomic abundance matrix was used for all downstream diversity, community composition, functional, and statistical analyses. Alpha diversity indices, including the Chao1 richness index, were calculated from this species-level taxonomic abundance matrix rather than directly from raw sequencing reads.

### 2.4. Statistical Analysis

All data processing, statistical analyses, and visualizations were performed in RStudio (v2025.09.0 + 387, Posit Software, PBC, Boston, MA, USA). Species-level bacterial and fungal abundance tables obtained from shotgun metagenomic sequencing were filtered to retain only reliably identified taxa.

Soil physicochemical properties, including pH, Arany plasticity index, water-soluble salts (WSS), CaCO_3_, humus, P_2_O_5_, K_2_O, and nitrate concentration, were compared among land-use systems using Kruskal–Wallis tests followed by Dunn’s post hoc comparisons. Alpha and beta diversity analyses were performed separately for bacterial and fungal communities using species-level taxonomic abundance matrices. Alpha diversity was evaluated using the Chao1 richness and Shannon diversity indices. Differences among land-use types and soil depths were assessed using Kruskal–Wallis tests followed by Dunn’s post hoc comparisons with Benjamini–Hochberg (BH) adjustment, whereas topographic position and plant system were compared using Wilcoxon rank-sum tests. Land-use type and plant system analyses were restricted to topsoil samples (0–30 cm), while soil-depth analyses included only feed corn and sweet corn samples collected at 0–30, 31–60, and 61–90 cm. Relationships between alpha diversity and soil physicochemical properties were evaluated separately for bacterial and fungal communities using Spearman’s rank correlation analysis with Benjamini–Hochberg (BH)-adjusted *p*-values. Correlation coefficients were visualized as bubble plots, where bubble size represented the absolute Spearman correlation coefficient (|ρ|) and bubble colour indicated the direction and magnitude of the correlation. Beta diversity was assessed separately for bacterial and fungal communities using Bray–Curtis dissimilarities and visualized by non-metric multidimensional scaling (NMDS). Differences in community composition were evaluated using PERMANOVA (999 permutations), while homogeneity of multivariate dispersion was assessed using the betadisper procedure. Both presence/absence and abundance-based approaches were used to evaluate shared taxa, dominant species, and differences among land-use systems, soil depths, and environmental gradients. Bacterial and fungal community composition was further examined through land-use-specific comparisons and species overlap analyses.

To investigate ecological functions, bacterial and fungal taxa identified by shotgun metagenomic sequencing were assigned to twelve ecological functional groups based on published evidence describing the ecological roles of individual microbial taxa [16]. The functional groups included nitrogen fixation, phosphorus solubilization, potassium solubilization, plant growth promotion, phytohormone production, siderophore production, mycorrhizal symbiosis, organic matter decomposition, methane oxidation, hydrocarbon degradation, bioremediation, and antibiotic resistance. Taxa reported to perform the same ecological function were grouped into the corresponding functional category for downstream analyses. Functional community analyses were performed using only topsoil samples (0–30 cm depth). For each functional group, Bray–Curtis dissimilarity matrices were calculated from species-level abundance data and visualized using non-metric multidimensional scaling (NMDS). Overall differences among land-use systems were evaluated using permutational multivariate analysis of variance (PERMANOVA; 999 permutations). Pairwise comparisons between land-use systems were subsequently performed using pairwise PERMANOVA, with *p*-values adjusted using the Benjamini–Hochberg false discovery rate (FDR) procedure. Mean pairwise Bray–Curtis dissimilarities among land-use systems were additionally visualized as bubble plots, where bubble size and colour intensity were proportional to community dissimilarity, and statistically significant pairwise comparisons (adjusted *p* < 0.05) were indicated by an asterisk. Depth-related variation in microbial functional potential was further evaluated using shotgun metagenomic functional profiles. Functional pathway abundance analyses were based on the processed KEGG annotation dataset generated from the shotgun metagenomic sequencing data. KEGG orthologs were assigned to their corresponding metabolic pathways, and the resulting KEGG pathway abundance values were used for downstream statistical analyses and visualization. These values represent functional pathway abundance rather than taxonomic relative abundance. Oxidative stress-related KEGG orthologs were extracted and grouped into five literature-based categories: Reactive Oxygen Species Detoxification (ROS), Redox Homeostasis, Oxidative Damage Repair, Stress Regulation, and Antioxidant Metabolite Production (Supplementary Materials (Table S1)) [17,18,19,20,21,22,23,24,25,26,27,28,29,30,31,32,33,34,35,36,37,38,39,40,41,42,43]. Functional differences among soil depths were evaluated using Bray–Curtis dissimilarities and Principal Coordinate Analysis (PCoA), while statistical significance was assessed using PERMANOVA and Wilcoxon rank-sum tests. To identify oxidative stress-related biomarkers associated with topsoil and subsoil communities, Linear Discriminant Analysis Effect Size (LEfSe) was performed using normalized KEGG pathway abundance values. Significantly enriched KEGG orthologs (adjusted *p* < 0.05) were manually classified into five literature-based oxidative stress-related functional categories. For visualization, significant biomarkers were ordered according to functional category and descending LDA score and displayed using a circular lollipop plot. The relative contribution of Topsoil- and Subsoil-enriched biomarkers within each functional category was additionally summarized using donut charts showing the proportion and total number of significant KEGG orthologs assigned to each category. In addition, selected KEGG pathways related to carbon, nitrogen, phosphorus, sulfur, and iron metabolism were compared between topsoil and subsoil using Wilcoxon rank-sum tests. The complete LEfSe output is provided in Supplementary Materials (Table S2).

Microbial dominance structure was evaluated using treatment-specific mean relative abundance thresholds. Taxa with relative abundances above the treatment-specific mean were classified as dominant, whereas those below the mean were considered rare. Microbial interactions were investigated through co-occurrence network analyses and keystone taxa identification. Network topology metrics, including node connectivity and modularity, were used to compare microbial interaction patterns among land-use systems and soil depths.

Bacterial and fungal species with reported phytopathogenic potential were identified based on published evidence of phytopathogenicity using literature-based species lists developed separately for crop (feed corn and sweet corn) [44,45,46,47,48,49,50,51,52,53] and orchard (quince and walnut) systems [54,55,56,57,58,59,60,61,62,63,64,65,66,67,68,69,70,71,72,73,74]. The complete species lists are provided in the Supplementary Materials (Table S3). For pathogen composition analyses, bacterial and fungal species with reported phytopathogenic potential were ranked according to their mean relative abundance across all samples within each production system (crops or orchards). The most abundant species with reported phytopathogenic potential were subsequently selected for visualization. Relative abundance values presented in stacked bar plots were normalized to 100% within the selected subset of species with reported phytopathogenic potential and therefore represent the relative composition of this selected subset rather than the entire soil microbiome. The prevalence, composition, abundance patterns, vertical distribution, and relationships of species with reported phytopathogenic potential with soil physicochemical properties were evaluated using prevalence analyses, abundance profiling, and Pearson correlation analysis.

Statistical significance was defined at *p* < 0.05. Data manipulation was performed in R using the dplyr, tidyr, and stringr packages; ecological analyses were conducted using vegan and Hmisc; network analyses were performed using NetCoMi; statistical testing was carried out using rstatix; LEfSe analyses were performed using microbiomeMarker. Graphical visualizations, including circular lollipop plots, donut charts, heatmaps, and Venn diagrams, were generated using ggplot2, patchwork, pheatmap, and VennDiagram.

## 3. Results

### 3.1. Soil Physicochemical Properties Across Land-Use Systems

**Figure 2:**
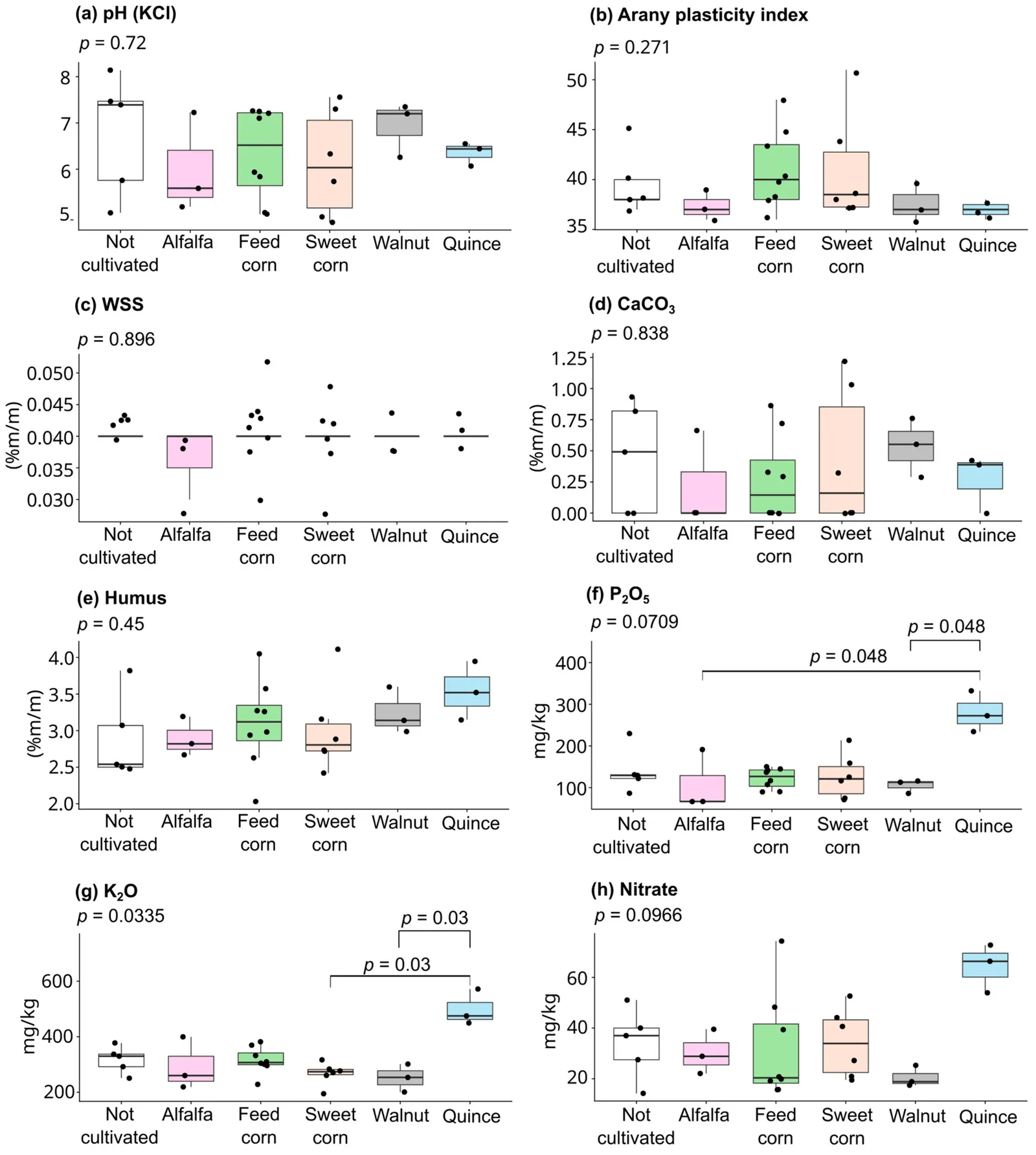
Boxplots of soil physicochemical properties across six land-use systems based on topsoil samples (0–30 cm). Panels (**a**–**h**) show pH (KCl), Arany plasticity index, water-soluble salts (WSS), CaCO_3_, humus, available P_2_O_5_, available K_2_O, and nitrate concentration, respectively. Boxplots show the median, interquartile range, whiskers, and individual observations. Overall differences among land-use systems were assessed using the Kruskal–Wallis test, and significant pairwise comparisons identified by Dunn’s post hoc test are indicated.

The evaluated land-use systems exhibited generally similar soil physicochemical properties, although several nutrient-related parameters showed noticeable variation (Figure 2). Soil pH ranged from approximately 5.0 to 8.2, with median values between 5.6 and 7.2, and did not differ significantly among treatments (*p* = 0.72). Likewise, the Arany plasticity index (36–51; *p* = 0.271), WSS (0.028–0.051%; *p* = 0.896), CaCO_3_ content (0–1.2%; *p* = 0.838), humus content (2.0–4.1%; *p* = 0.45), and nitrate concentration (15–72 mg kg^−1^; *p* = 0.0966) remained relatively consistent across land-use categories.

In contrast, greater variability was observed for the plant-available nutrient fractions. Available phosphorus (P_2_O_5_) ranged from approximately 70 to 330 mg kg^−1^ and showed a marginal trend toward differences among land-use systems (Kruskal–Wallis, *p* = 0.0709). Although the overall test was not statistically significant, pairwise comparisons suggested higher P_2_O_5_ concentrations in quince soils than in sweet corn and walnut soils (adjusted *p* = 0.048). Pairwise comparisons indicated higher P_2_O_5_ concentrations in quince soils than in sweet corn and walnut soils (adjusted *p* = 0.048). Available potassium (K_2_O) exhibited the strongest land-use response (*p* = 0.0335), with the highest concentrations recorded in quince soils (approximately 450–580 mg kg^−1^). Dunn’s post hoc test further revealed significantly higher K_2_O values in quince soils compared with sweet corn and walnut soils (adjusted *p* = 0.03).

### 3.2. Soil Microbial Diversity

**Figure 3:**
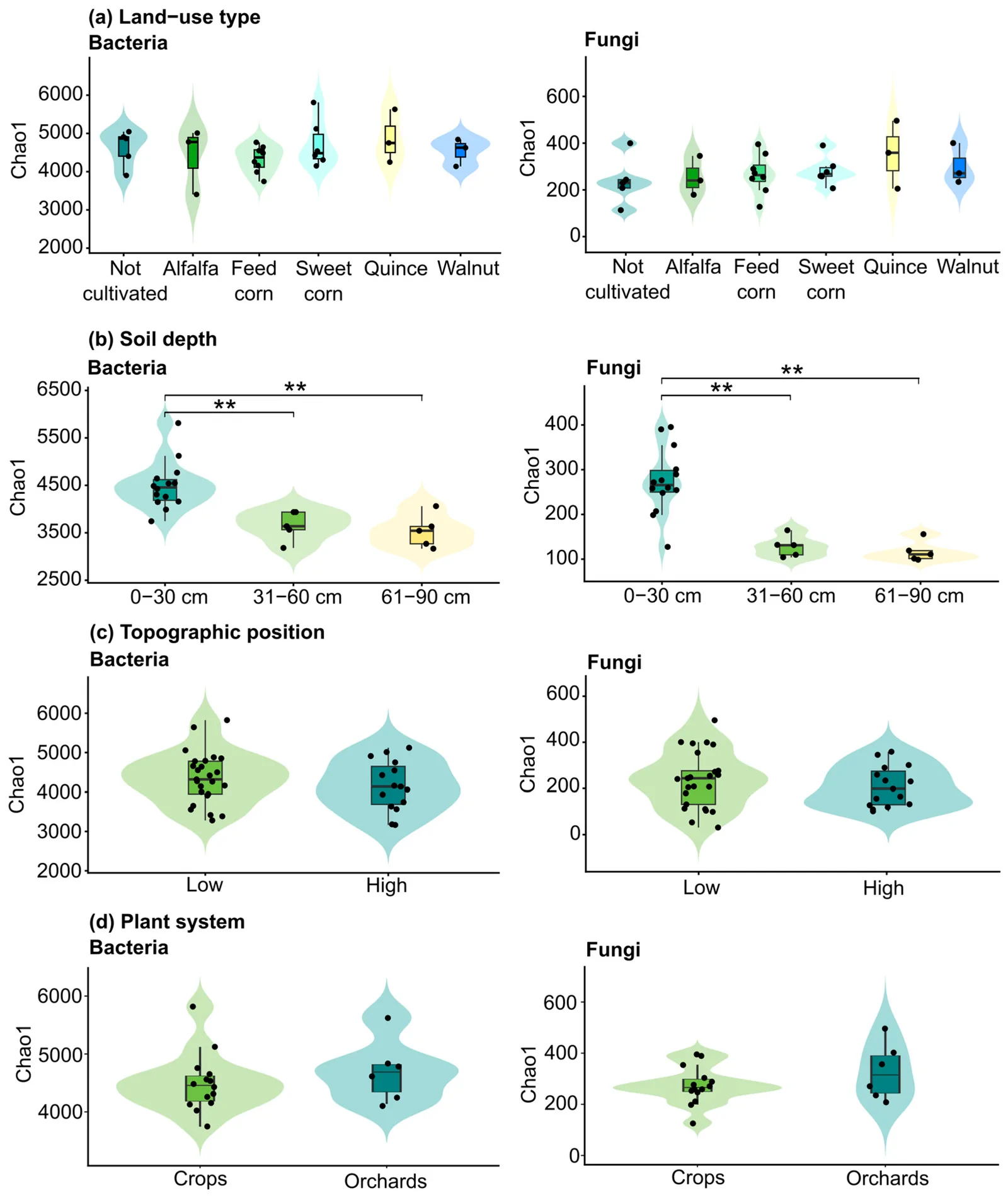
Alpha diversity of bacterial and fungal soil microbial communities assessed using the Chao1 richness index across different environmental and management factors. Panels show comparisons among land-use types in the 0–30 cm soil layer (**a**), soil depths (**b**), topographic positions (**c**), and plant systems (**d**). Violin plots (shaded areas) illustrate the distribution and density of observations, boxplots indicate the median and interquartile range, and points represent individual soil samples. Land-use type and plant system analyses were performed using only topsoil samples (0–30 cm). Soil-depth comparisons were performed exclusively using feed corn and sweet corn samples collected from the 0–30, 31–60, and 61–90 cm soil layers. Plant system comparisons included only crop (feed corn and sweet corn) and orchard (quince and walnut) samples collected from the 0–30 cm soil layer, whereas alfalfa and not cultivated soils were excluded from this comparison. Topographic-position comparisons included all available samples. Statistical significance was evaluated using Kruskal–Wallis tests for multiple-group comparisons, followed by Dunn’s post hoc test where appropriate, whereas Wilcoxon rank-sum tests were applied for comparisons between two groups. Double asterisks (**) indicate statistically significant differences (*p* < 0.01).

Chao1 richness exhibited contrasting responses across the evaluated environmental and management factors (Figure 3). Within the 0–30 cm soil layer, neither bacterial nor fungal richness differed significantly among land-use types (Kruskal–Wallis, *p* > 0.05; Figure 3a), although bacterial richness tended to be higher in quince and sweet corn soils, whereas fungal richness was highest in quince soils. In contrast, soil depth significantly influenced bacterial and fungal richness (Figure 3b). For bacteria, the overall effect of depth was significant (Kruskal–Wallis, χ^2^ = 15.03, *p* = 0.00054). Dunn’s post hoc analysis showed significantly higher richness in the 0–30 cm layer than in the 31–60 cm (adjusted *p* = 0.0059) and 61–90 cm layers (adjusted *p* = 0.0033), whereas no significant difference was detected between the two deeper layers (adjusted *p* = 0.7542). A similar pattern was observed for fungi, where Chao1 richness also differed significantly among soil depths (Kruskal–Wallis, χ^2^ = 15.18, *p* = 0.00051). Pairwise comparisons indicated significantly greater richness in the 0–30 cm layer than in both the 31–60 cm (adjusted *p* = 0.0083) and 61–90 cm layers (adjusted *p* = 0.0023), while the two subsurface layers did not differ significantly (adjusted *p* = 0.6228). Chao1 richness was not significantly affected by topographic position (Figure 3c) or by plant system (Figure 3d). Although orchard soils exhibited slightly higher richness than crop soils, these differences were not statistically significant (Wilcoxon test, *p* = 0.444 for both bacterial and fungal communities).

**Figure 4:**
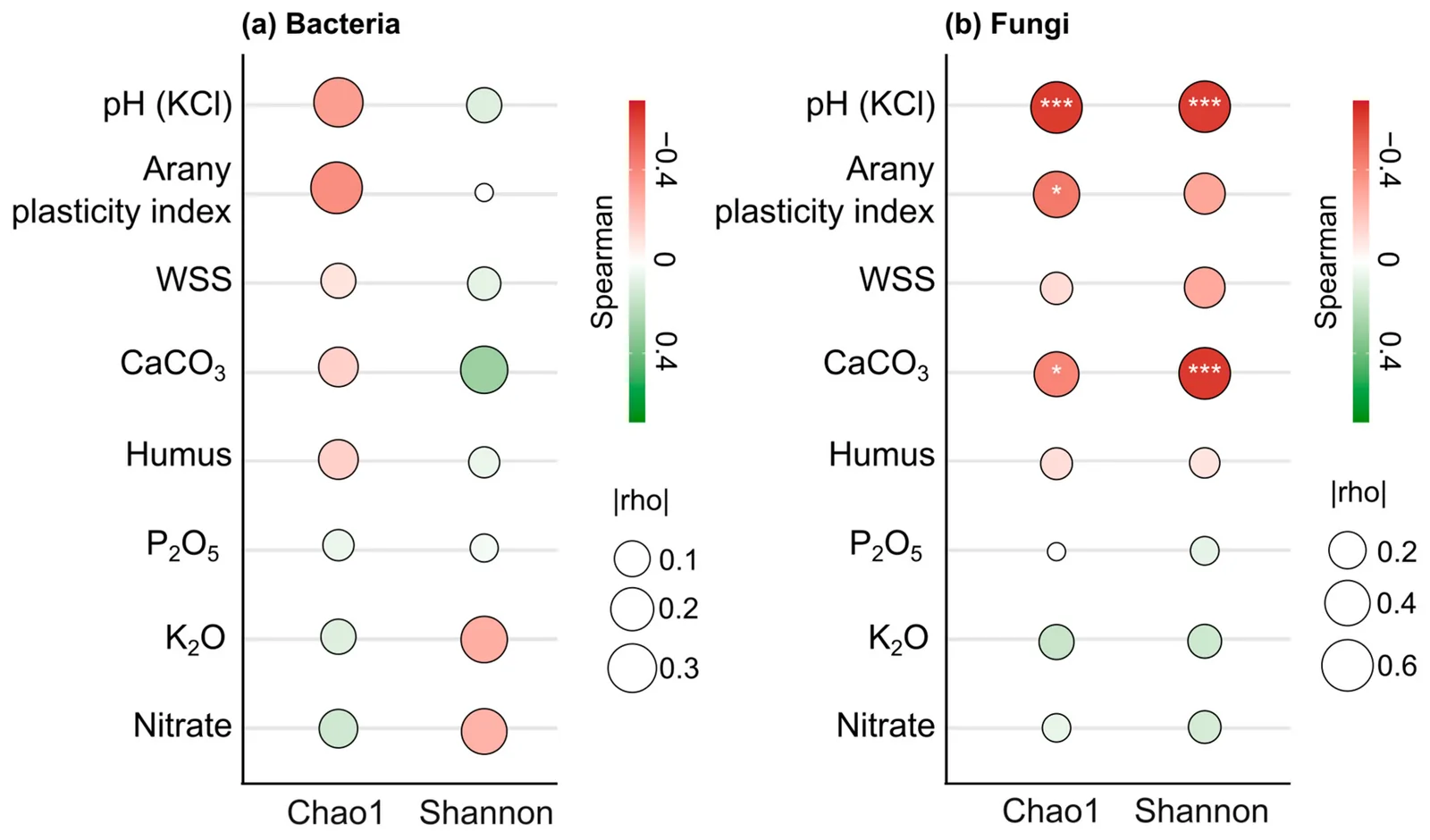
Relationships between soil physicochemical properties and bacterial (**a**) and fungal (**b**) alpha diversity assessed using the Chao1 richness and Shannon diversity indices. Correlations were evaluated separately for bacterial and fungal communities using Spearman’s rank correlation analysis with Benjamini–Hochberg (BH)-adjusted *p*-values. Bubble size represents the absolute value of the Spearman correlation coefficient (|ρ|), whereas bubble colour indicates the direction and magnitude of the correlation, with positive correlations shown in green and negative correlations in red. Statistically significant correlations after BH correction are indicated by asterisks (*, adjusted *p* < 0.05; ***, adjusted *p* < 0.001).

Relationships between soil physicochemical properties and microbial alpha diversity differed markedly between bacterial and fungal communities (Figure 4). Bacterial alpha diversity exhibited only weak to moderate correlations with the measured soil properties, and none remained statistically significant after Benjamini–Hochberg correction. Bacterial Chao1 richness showed the strongest negative associations with the Arany plasticity index (ρ = −0.38) and soil pH (ρ = −0.33), whereas Shannon diversity was weakly positively associated with CaCO_3_ (ρ = 0.29) and negatively associated with K_2_O (ρ = −0.27), although all adjusted *p*-values exceeded 0.05. Fungal alpha diversity displayed stronger relationships with soil physicochemical properties. Fungal Chao1 richness was significantly negatively correlated with soil pH (ρ = −0.63, adjusted *p* = 0.00010), the Arany plasticity index (ρ = −0.45, adjusted *p* = 0.0143), and CaCO_3_ (ρ = −0.41, adjusted *p* = 0.0233). Likewise, Shannon diversity was strongly negatively correlated with soil pH (ρ = −0.63, adjusted *p* = 5.79 × 10^−5^) and CaCO_3_ (ρ = −0.64, adjusted *p* = 5.79 × 10^−5^), whereas correlations with the remaining soil properties were weak and not statistically significant.

### 3.3. Soil Microbial Community Composition

**Figure 5:**
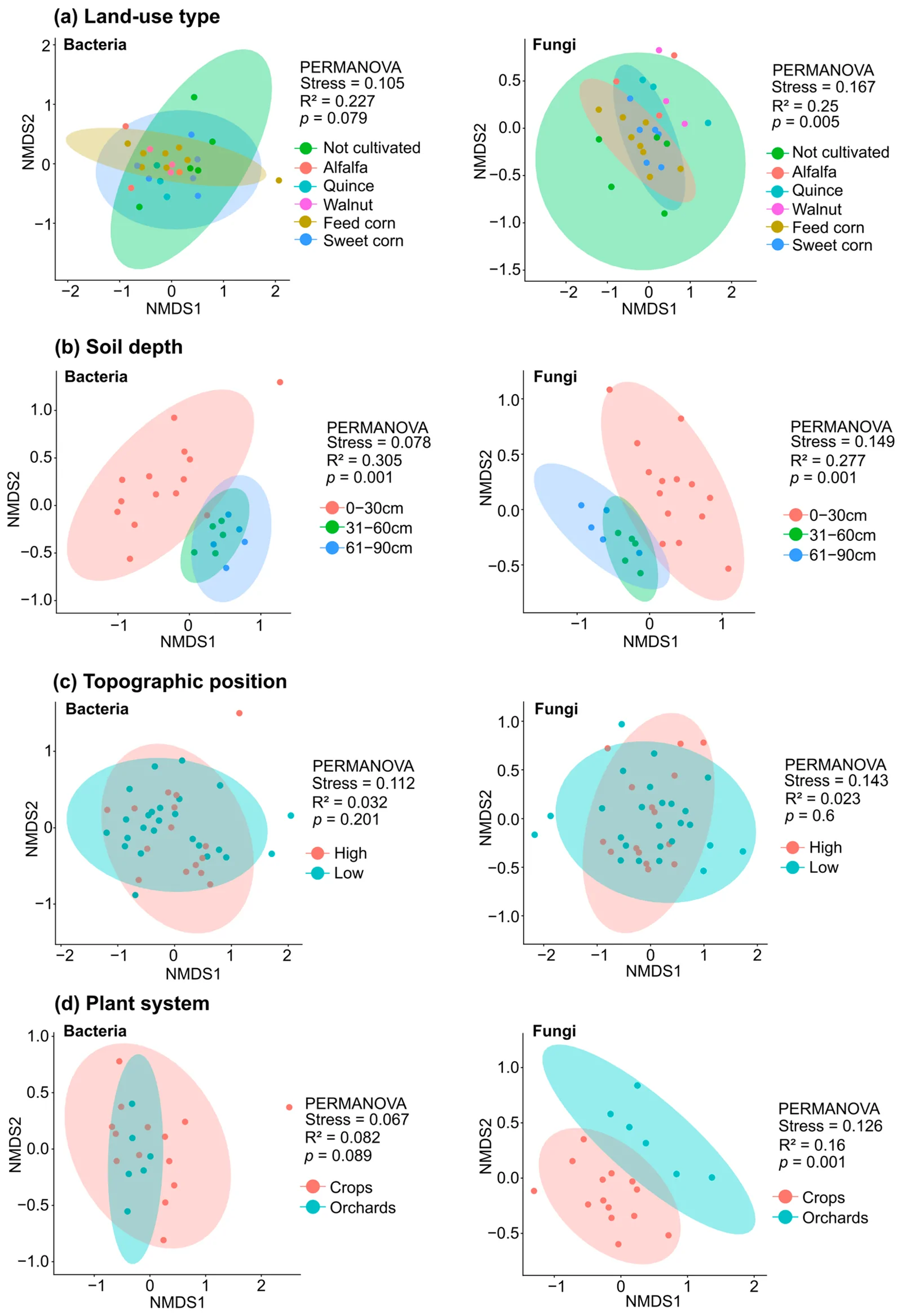
Non-metric multidimensional scaling (NMDS) ordination of bacterial (**left**) and fungal (**right**) community composition based on Bray–Curtis dissimilarities calculated from species-level abundance data. Panels show differences among land-use types in the 0–30 cm soil layer (**a**), soil depths (**b**), topographic positions (**c**), and plant systems (**d**). Land-use type and plant system analyses were restricted to topsoil samples (0–30 cm), whereas soil-depth analyses included only feed corn and sweet corn samples collected at 0–30, 31–60, and 61–90 cm. Ellipses represent the 95% confidence intervals around group centroids. NMDS stress values and PERMANOVA results (R^2^ and *p*-values) are shown within each panel.

Non-metric multidimensional scaling (NMDS) revealed distinct patterns of bacterial and fungal community composition across the evaluated environmental and management factors (Figure 5). Among land-use types, bacterial communities showed moderate separation among groups, although differences were not statistically significant (PERMANOVA, R^2^ = 0.227, *p* = 0.079; Figure 5a). In contrast, fungal community composition differed significantly among land-use types (R^2^ = 0.250, *p* = 0.005). Soil depth was the strongest factor structuring both bacterial and fungal communities (Figure 5b). Bacterial community composition differed significantly among soil layers (PERMANOVA, R^2^ = 0.305, *p* = 0.001), with clear separation of topsoil (0–30 cm) from the deeper layers in the NMDS ordination. A similar pattern was observed for fungi, where soil depth also explained a substantial proportion of community variation (R^2^ = 0.277, *p* = 0.001). In contrast, topographic position had little influence on microbial community composition (Figure 5c). Neither bacterial (R^2^ = 0.032, *p* = 0.201) nor fungal communities (R^2^ = 0.023, *p* = 0.600) differed significantly between high and low topographic positions. Differences between crop (feed corn and sweet corn) and orchard (quince and walnut) systems were also community-dependent (Figure 5d). Bacterial community composition did not differ significantly between production systems (R^2^ = 0.082, *p* = 0.089). In contrast, fungal communities exhibited clear separation between crop and orchard soils, with plant system explaining 16.0% of the observed variation (PERMANOVA, R^2^ = 0.160, *p* = 0.001).

### 3.4. Land-Use Effects on Bacterial and Fungal Assemblages

**Figure 6:**
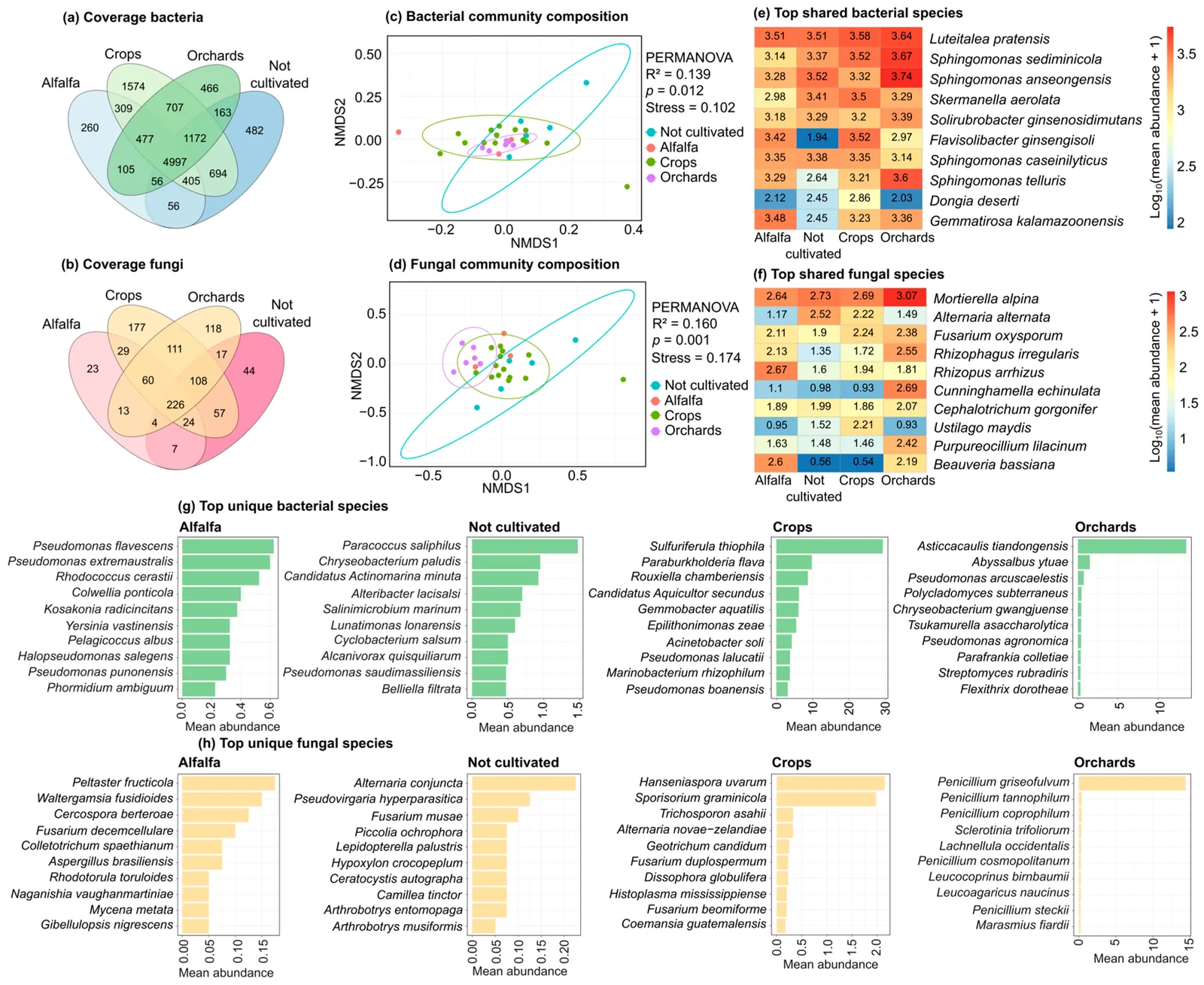
Community structure of bacterial and fungal assemblages across four land-use systems (alfalfa, not cultivated land, crops, and orchards) in topsoil samples (0–30 cm depth). Panels (**a**,**b**) show coverage-based analyses, including Venn diagrams of shared and unique bacterial and fungal species among land-use systems. Panels (**c**,**d**) present NMDS ordinations based on Bray–Curtis dissimilarities calculated from species abundance data for bacterial and fungal communities, respectively. Ellipses indicate group dispersion and PERMANOVA statistics are reported within each panel. Panels (**e**,**f**) display heatmaps of the ten most abundant bacterial and fungal species shared among all land-use systems, selected by ranking species according to their mean abundance across all samples. For the shared-community heatmaps, the minimum mean abundance of the selected Top 10 species was 307.93 for bacteria and 54.28 for fungi. Colors represent mean log10-transformed abundance values. Panels (**g**,**h**) show the ten most abundant unique bacterial and fungal species detected exclusively within each land-use system, selected according to their mean abundance within the corresponding land-use system. Bar lengths represent mean species abundance.

Land-use systems influenced both the composition and distribution of bacterial and fungal communities in topsoil (Figure 6). Coverage-based analyses revealed the presence of a substantial shared microbial core among all land-use systems, particularly for bacteria. A total of 4997 bacterial species were detected across alfalfa, not cultivated, crop, and orchard soils (Figure 6a), representing the dominant fraction of the bacterial community. Nevertheless, each land-use system also harbored unique bacterial taxa, with crops containing the highest number of unique species (1574), followed by not cultivated soils (482), orchards (466), and alfalfa soils (260).

Fungal communities exhibited a smaller shared core and a greater proportion of unique taxa (Figure 6b). Only 226 fungal species were common to all land-use systems, whereas unique species represented a substantially larger component of the fungal assemblage. Crops again contained the highest number of unique fungal species (177), followed by orchards (118), not cultivated soils (44), and alfalfa soils (23).

The differences observed in species occurrence were further reflected in overall community composition. NMDS ordinations demonstrated significant separation among land-use systems for both bacterial and fungal assemblages. Bacterial communities differed significantly among land-use categories (PERMANOVA, R^2^ = 0.139, *p* = 0.012; Figure 6c), with land-use explaining approximately 14% of the variation in community structure. Fungal communities exhibited an even stronger response (R^2^ = 0.160, *p* = 0.001; Figure 6d).

The abundance patterns of dominant shared species further highlighted these compositional differences (Figure 6e,f). Among bacteria, members of the genera *Sphingomonas*, *Skermanella*, *Flavisolibacter*, and *Gemmatirosa* displayed contrasting abundance profiles across land-use systems. Similarly, dominant fungal species such as *Mortierella alpina*, *Alternaria alternata*, *Fusarium oxysporum*, *Rhizophagus irregularis*, and *Ustilago maydis* varied substantially among treatments.

Analysis of unique taxa provided additional evidence for land-use-specific microbial signatures (Figure 6g,h). Crop soils were characterized by highly abundant unique bacterial species, including *Sulfuriferula thiophila* and *Paraburkholderia lava*, whereas orchard soils contained characteristic taxa such as *Asticcacaulis tiandongensis*. Likewise, fungal communities exhibited distinct land-use-associated species, with *Hanseniaspora uvarum* dominating crop-specific assemblages and *Penicillium griseofulvum* representing the most abundant orchard-specific fungal species.

### 3.5. Microbial Dominance Structure and Community Evenness

To determine whether the observed taxonomic and functional differences among land-use systems were driven by a small number of dominant taxa or reflected broader community-wide shifts, species were classified into above-average and below-average groups based on treatment-specific mean relative abundance thresholds.

**Figure 7:**
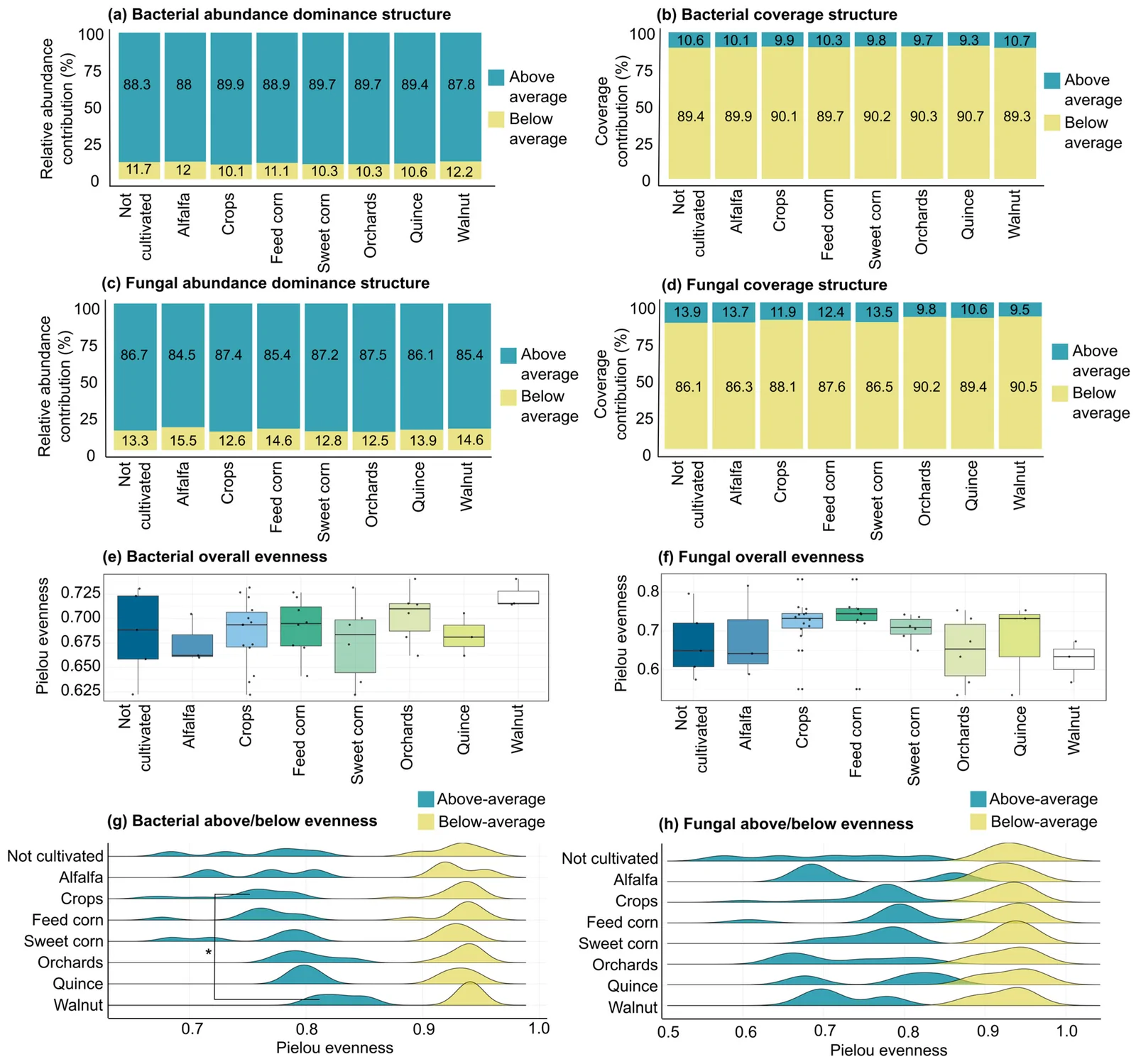
Dominance structure and community evenness of bacterial and fungal assemblages across different land-use systems based on species-level communities from topsoil samples (0–30 cm depth). Panels (**a**,**b**) show the contribution of above-average and below-average taxa to total bacterial and fungal abundance, respectively, whereas panels (**c**,**d**) present the corresponding contribution to species coverage. Taxa were classified as above-average or below-average according to treatment-specific mean relative abundance thresholds. Panels (**e**,**f**) display Pielou’s evenness values for overall bacterial and fungal communities, respectively. Panels (**g**,**h**) show ridge-density distributions of Pielou’s evenness for above-average and below-average taxa. Numbers within the circular plots correspond to land-use systems: 1 = Not cultivated, 2 = Alfalfa, 3 = Crops, 4 = Feed corn, 5 = Sweet corn, 6 = Orchards, 7 = Quince, and 8 = Walnut. Differences among groups were assessed using Kruskal–Wallis, tests followed by Dunn’s post hoc test with Benjamini–Hochberg correction for multiple comparisons. A single asterisk (*) indicates statistically significant differences (*p* < 0.05).

Across all land-use systems, a relatively small proportion of taxa accounted for most microbial abundance (Figure 7). In bacterial communities, above-average taxa contributed approximately 87–90% of total abundance, whereas below-average taxa accounted for only 10–13% (Figure 7a). A similar pattern was observed for fungi, where dominant taxa represented approximately 84–88% of total abundance (Figure 7b). In contrast, richness patterns showed the opposite trend. Above-average bacterial taxa represented only 10–15% of detected species, while below-average taxa accounted for approximately 85–90% of total richness (Figure 7c). Comparable distributions were observed for fungal communities (Figure 7d).

Despite the compositional differences identified in previous analyses, dominance structure remained remarkably consistent across land-use systems. Only minor variation was observed in the relative contribution of dominant and rare taxa.

This pattern was further supported by Pielou’s evenness values. Bacterial communities exhibited relatively uniform evenness across all systems, ranging from approximately 0.66 to 0.73 (Figure 7e). Fungal communities showed slightly greater variability, with median values ranging from approximately 0.62 to 0.75 (Figure 7f), although differences among land-use systems remained modest overall.

The ridge-density analyses revealed a clear separation between dominance classes (Figure 7g,h). In both bacterial and fungal communities, above-average taxa consistently displayed lower Pielou evenness values than below-average taxa. This pattern was particularly pronounced for fungi, where the distributions of the two dominance classes showed minimal overlap.

### 3.6. Microbial Network Organization

**Figure 8:**
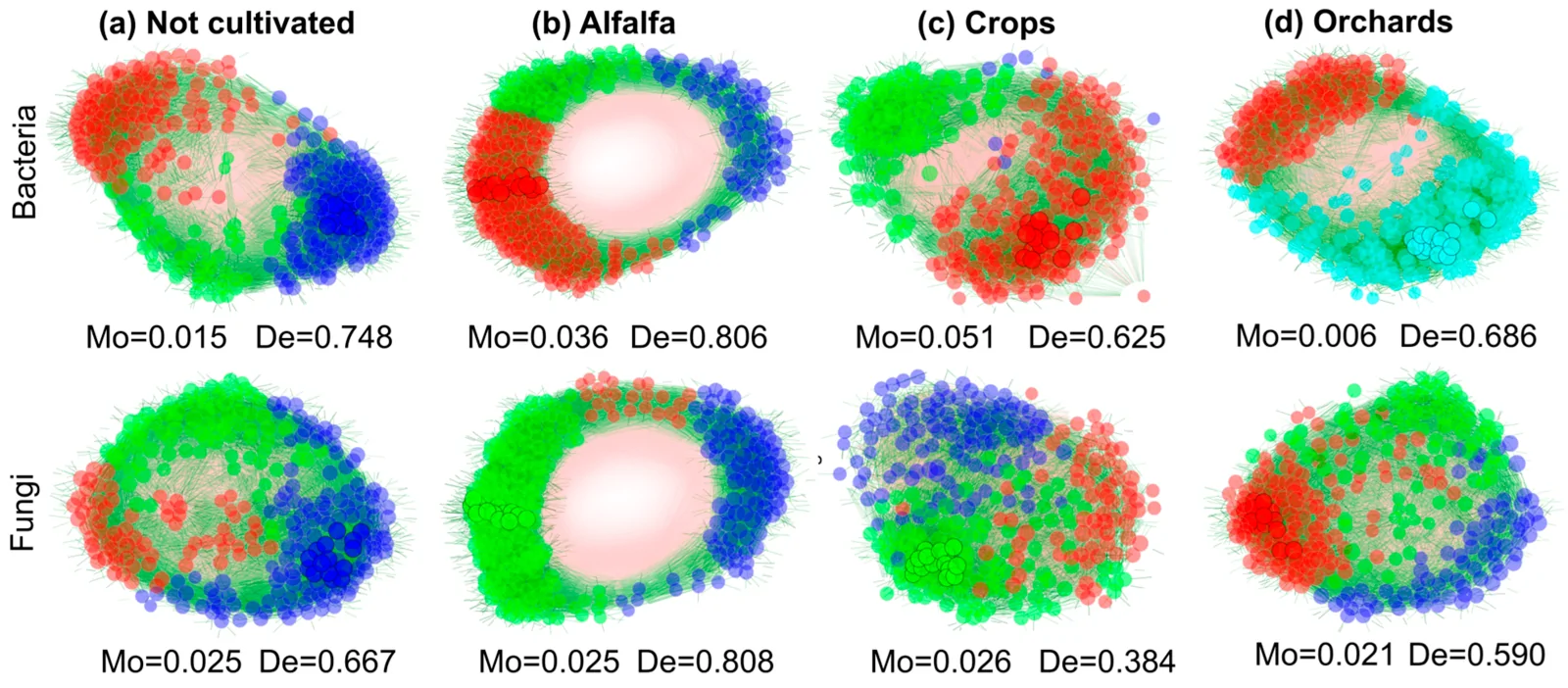
Co-occurrence network structure of bacterial and fungal communities across four land-use systems in topsoil samples (0–30 cm depth): not cultivated (**a**), alfalfa (**b**), crops (**c**), and orchards (**d**). Nodes represent microbial taxa and edges represent significant co-occurrence relationships. Node colors indicate network modules. Network modularity (Mo) and density (De) values are reported for each community. Higher density values indicate a greater degree of connectivity among taxa, whereas higher modularity values reflect a stronger subdivision of the network into distinct modules.

Although land-use systems exhibited broadly similar dominance structures and levels of community evenness, co-occurrence network analyses revealed clear differences in the organization and connectivity of microbial communities (Figure 8). Network density varied considerably among management systems.

Alfalfa soils exhibited the highest network density for both bacterial (De = 0.806) and fungal communities (De = 0.808). In contrast, crop soils displayed the lowest fungal network density (De = 0.384). Not cultivated soils also maintained relatively high connectivity for both bacteria (De = 0.748) and fungi (De = 0.667), whereas orchard soils exhibited intermediate density values (bacteria: De = 0.686; fungi: De = 0.590).

Compared with density, differences in modularity were less pronounced. Bacterial modularity ranged from 0.006 in orchards to 0.051 in crop soils, while fungal modularity varied only slightly between 0.021 and 0.026. Overall, all networks were characterized by relatively low modularity values.

The network topologies visually reflected these patterns. Alfalfa and not cultivated soils displayed densely connected bacterial and fungal communities with numerous links among taxa, whereas crop soils were characterized by reduced connectivity, particularly within fungal communities. Orchard soils occupied an intermediate position, maintaining relatively connected bacterial networks while supporting fungal communities that were less integrated than those observed in alfalfa soils.

**Figure 9:**
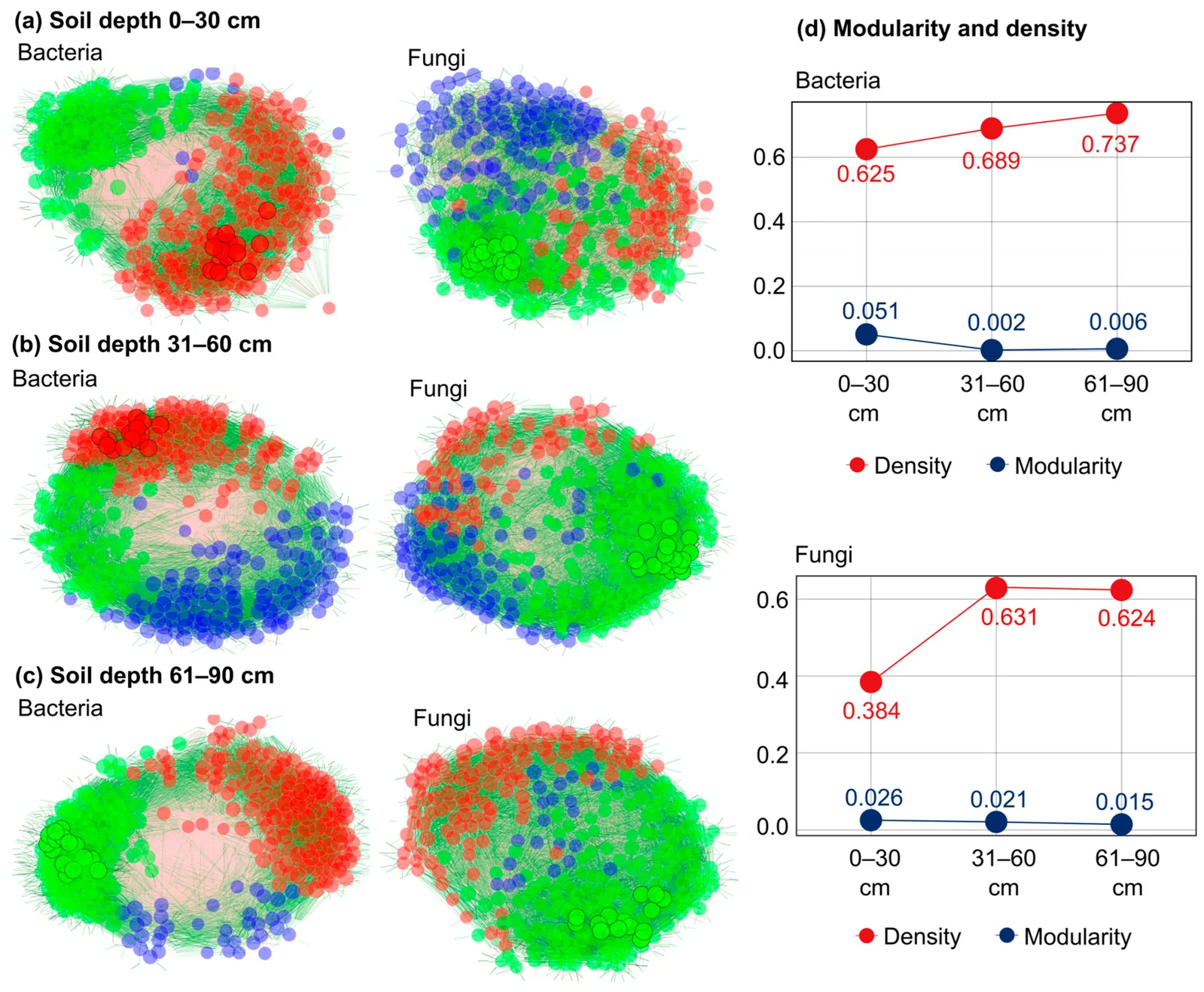
Co-occurrence network structure of bacterial and fungal communities across soil depths. Panels (**a**–**c**) show bacterial and fungal co-occurrence networks in the 0–30 cm, 31–60 cm, and 61–90 cm soil layers, respectively, using only feed corn and sweet corn samples collected across the three soil depths. Nodes represent microbial taxa and edges represent significant co-occurrence relationships. Node colors indicate network modules, while larger nodes highlight highly connected taxa within each network. Panel (**d**) summarizes network density and modularity values for bacterial and fungal communities across soil depths. Higher density values indicate greater connectivity among taxa, whereas higher modularity values reflect stronger subdivision of the network into distinct modules.

Given that soil depth emerged as the strongest driver of microbial community composition, co-occurrence networks were further examined across the three soil layers, using only feed corn and sweet corn samples, to determine whether vertical stratification also influenced microbial interaction patterns (Figure 9). Clear depth-related changes were observed in both bacterial and fungal networks. For bacteria, network density increased progressively from 0.625 in the 0–30 cm layer to 0.689 at 31–60 cm and 0.737 at 61–90 cm (Figure 9d). At the same time, bacterial modularity declined from 0.051 in the topsoil to 0.002 and 0.006 in the deeper layers.

Fungal communities exhibited a similar but more pronounced response. Network density increased substantially from 0.384 in the 0–30 cm layer to 0.631 at 31–60 cm and remained high at 0.624 in the deepest layer. The largest shift occurred between the topsoil and subsoil horizons. In contrast, fungal modularity remained consistently low, decreasing only slightly from 0.026 in the surface layer to 0.021 and 0.015 at greater depths.

The network visualizations supported these quantitative patterns. Fungal communities in the topsoil formed relatively sparse interaction networks, whereas both subsoil layers exhibited denser and more integrated structures. Bacterial communities displayed a comparable, although less pronounced, increase in connectivity with depth. Across all soil layers, modularity values remained low.

### 3.7. Land-Use Effects on Microbial Functional Composition

To facilitate ecological interpretation, the twelve literature-curated microbial functions were grouped into four broader ecosystem-function categories: Nutrient Cycling and Plant Nutrition, Plant–Microbe Interactions, Environmental Adaptation and Remediation, and Organic Matter Turnover and Carbon Cycling, following the classification framework previously developed and described by Gashi et al. [16].

**Figure 10:**
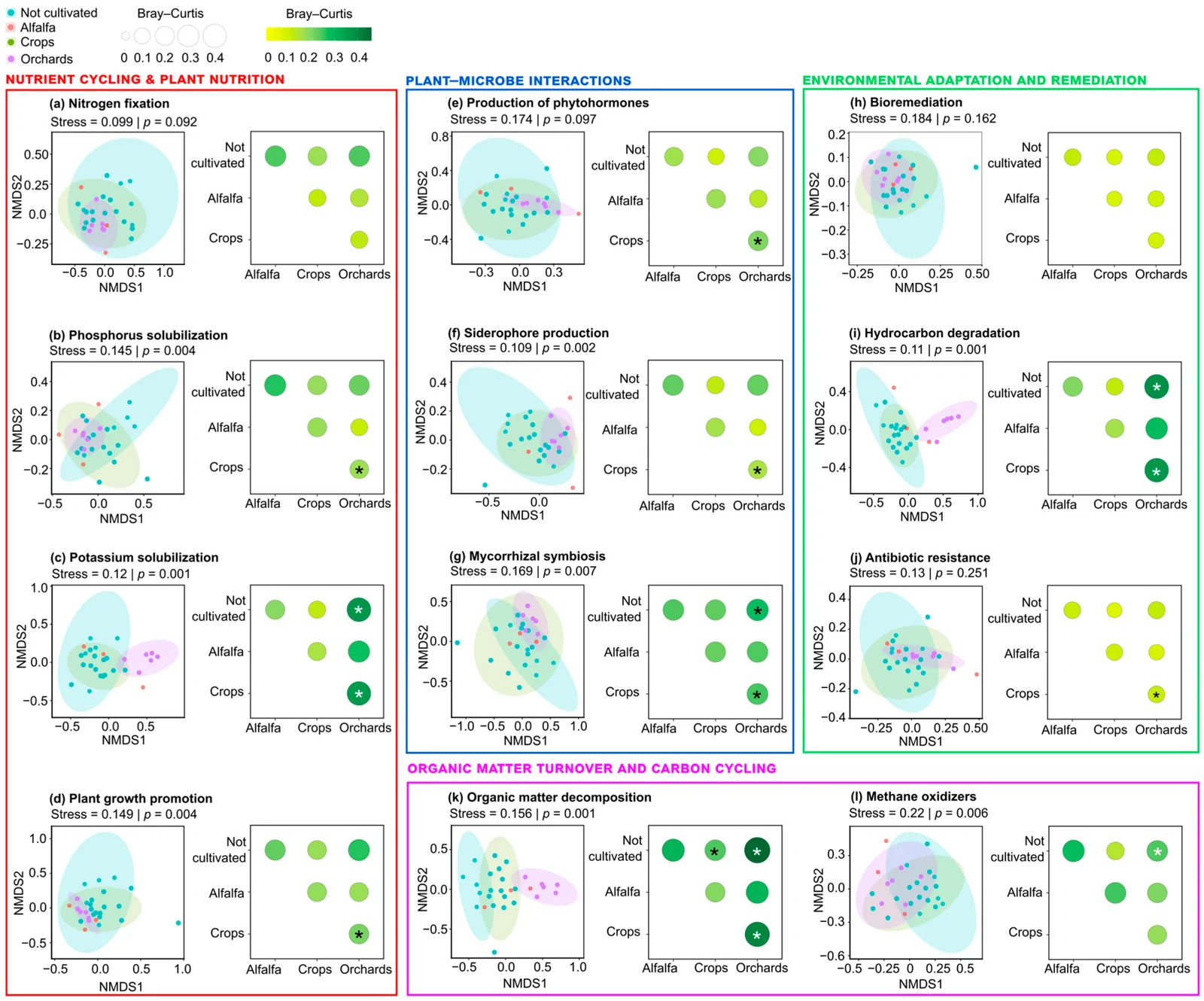
Functional differentiation of soil microbial communities across twelve ecological functions grouped into four broader ecosystem-function categories: Nutrient Cycling and Plant Nutrition, Plant–Microbe Interactions, Environmental Adaptation and Remediation, and Organic Matter Turnover and Carbon Cycling. For each functional group, the left panel presents a non-metric multidimensional scaling (NMDS) ordination based on Bray–Curtis dissimilarities calculated from function-associated microbial taxa detected in topsoil samples (0–30 cm). Points represent individual samples coloured according to land-use system (cultivated land, alfalfa, crops, and orchards), while shaded ellipses indicate group dispersion. Stress values and overall PERMANOVA *p*-values are reported within each NMDS panel. The corresponding bubble plots summarize the mean pairwise Bray–Curtis dissimilarities among land-use systems, where bubble size and colour intensity increase with increasing community dissimilarity. Asterisks (*) indicate statistically significant pairwise differences identified by pairwise PERMANOVA after Benjamini–Hochberg correction (adjusted *p* < 0.05).

Non-metric multidimensional scaling (NMDS) based on Bray–Curtis dissimilarities revealed that the composition of predicted microbial functional groups varied among land-use systems, although the degree of separation differed according to functional category (Figure 10). Within the Nutrient Cycling and Plant Nutrition category, phosphorus solubilization (PERMANOVA, *p* = 0.004, Figure 10b), potassium solubilization (*p* = 0.001, Figure 10c), and plant growth promotion (*p* = 0.004, Figure 10d) showed significant overall differences among land-use systems, whereas nitrogen fixation did not (*p* = 0.092, Figure 10a). Pairwise PERMANOVA further showed significant differences for phosphorus solubilization, potassium solubilization, and plant growth promotion after Benjamini–Hochberg correction, whereas no pairwise differences were detected for nitrogen fixation. Among Plant–Microbe Interactions, siderophore production (*p* = 0.002, Figure 10f) and mycorrhizal symbiosis (*p* = 0.007, Figure 10g) exhibited significant land-use-dependent clustering, while production of phytohormones showed only moderate overall separation (*p* = 0.097, Figure 10e). Pairwise analyses confirmed significant differences for siderophore production, mycorrhizal symbiosis, and production of phytohormones between selected land-use systems. Within the Environmental Adaptation and Remediation category, hydrocarbon degradation displayed one of the strongest separations among land-use systems (*p* = 0.001, Figure 10i), whereas bioremediation (*p* = 0.162, Figure 10h) and antibiotic resistance (*p* = 0.251, Figure 10j) exhibited substantial overlap among groups. Consistently, pairwise PERMANOVA identified significant differences only for hydrocarbon degradation, while bioremediation and antibiotic resistance remained non-significant after multiple-testing correction. Similarly, functions related to Organic Matter Turnover and Carbon Cycling differed significantly among land-use systems, as both organic matter decomposition (*p* = 0.001, Figure 10k) and methane oxidizers (*p* = 0.006, Figure 10l) exhibited distinct ordination patterns, particularly separating orchard-associated communities from the remaining land-use systems. Pairwise comparisons further revealed the highest number of significant differences for organic matter decomposition, whereas methane oxidizers showed a more limited but still significant differentiation among selected land-use systems.

### 3.8. Depth-Dependent Variation in Microbial Functional Potential

#### 3.8.1. Oxidative Stress-Related Functional Profiles

**Figure 11:**
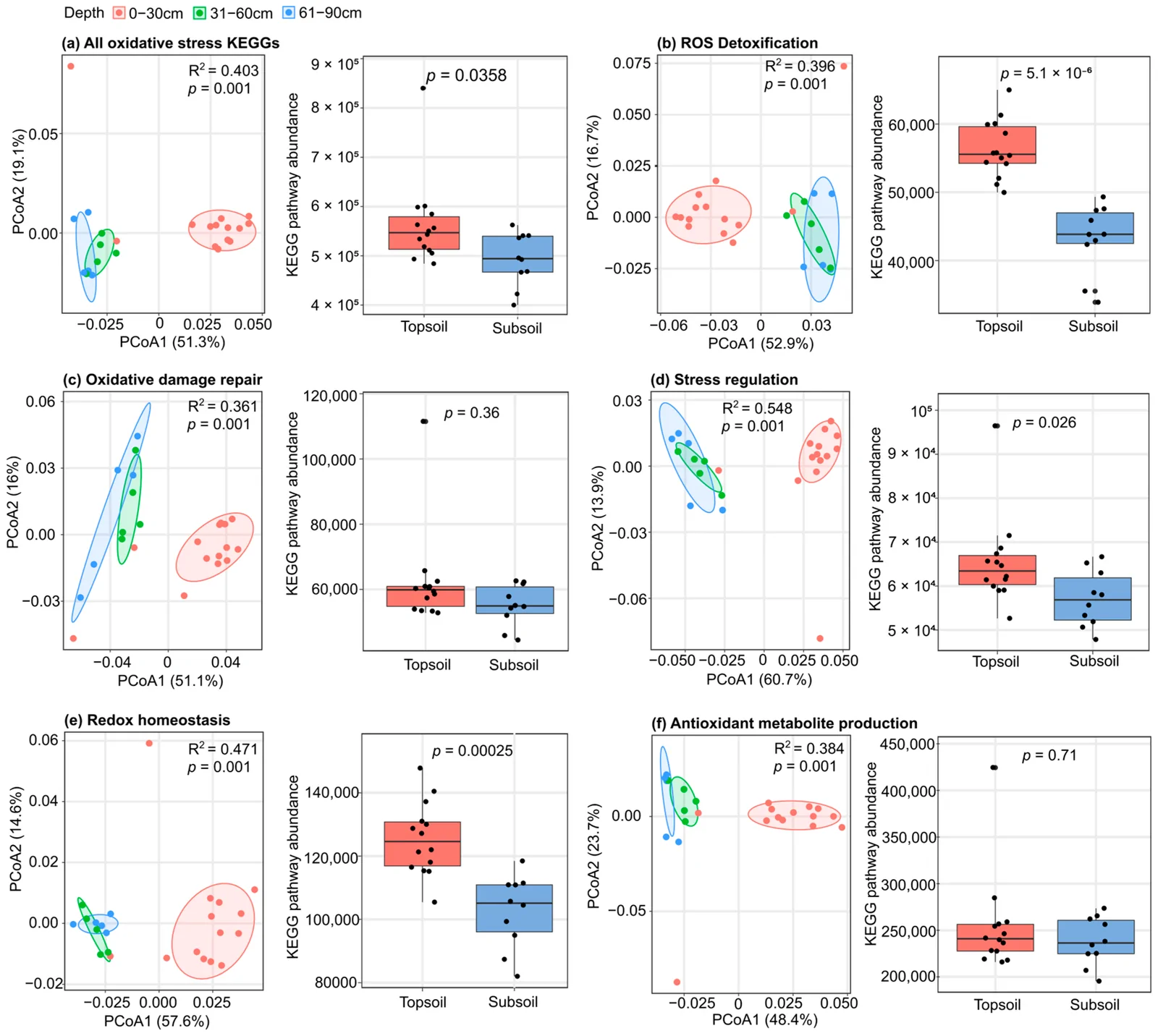
Depth-dependent variation in oxidative stress-related functional profiles in crop soils (feed corn and sweet corn) based on KEGG annotations. Oxidative stress genes were grouped into five functional categories: Reactive Oxygen Species Detoxification (ROS), Redox Homeostasis, Oxidative Damage Repair, Stress Regulation, and Antioxidant Metabolite Production. A detailed description of the functional grouping is provided in Supplementary Material (Table S1). Principal Coordinate Analysis (PCoA) based on Bray–Curtis dissimilarity illustrates the compositional differences among soil depths (0–30 cm, 31–60 cm, and 61–90 cm), while boxplots compare the corresponding functional potential between topsoil (0–30 cm) and subsoil (31–90 cm). Community-level differences were assessed using PERMANOVA, and differences in functional potential were evaluated using the Wilcoxon rank-sum test.

Oxidative stress-related microbial functions play a central role in protecting soil microorganisms from reactive oxygen species (ROS), maintaining intracellular redox balance, repairing oxidative damage, and regulating cellular responses to environmental stress. To facilitate ecological interpretation, oxidative stress-associated KEGG functions were grouped into five categories: ROS Detoxification, Redox Homeostasis, Oxidative Damage Repair, Stress Regulation, and Antioxidant Metabolite Production (Figure 11). These categories encompass the major microbial mechanisms involved in oxidative stress tolerance and adaptation, providing a functional framework for evaluating depth-dependent responses. The analysis revealed a clear vertical stratification of oxidative stress functions throughout the soil profile. When all oxidative stress-related KEGGs were considered together, microbial communities were significantly separated according to depth (R^2^ = 0.403, *p* = 0.001), and topsoil exhibited a significantly greater overall oxidative stress functional potential than subsoil (*p* = 0.0358) (Figure 11a).

Among the individual categories, Stress Regulation showed the strongest depth-related structuring (R^2^ = 0.548, *p* = 0.001) (Figure 11d), followed by Redox Homeostasis (R^2^ = 0.471, *p* = 0.001) (Figure 11e) and ROS Detoxification (R^2^ = 0.396, *p* = 0.001) (Figure 11b). Moreover, the functional potential of ROS Detoxification was substantially higher in topsoil than in subsoil (*p* = 5.1 × 10^−6^). Similarly, Redox Homeostasis functions were significantly enriched in topsoil (*p* = 0.00025) (Figure 11e). Stress Regulation functions also showed significantly greater potential in topsoil (*p* = 0.026).

In contrast, Oxidative Damage Repair displayed significant compositional differences across depths (R^2^ = 0.361, *p* = 0.001) but no significant difference in total functional potential between topsoil and subsoil (*p* = 0.36) (Figure 11c). Likewise, Antioxidant Metabolite Production exhibited clear depth-dependent community structuring (R^2^ = 0.384, *p* = 0.001), yet its overall functional potential remained comparable between soil layers (*p* = 0.71) (Figure 11f).

#### 3.8.2. LEfSe Biomarkers of Oxidative Stress Functions

**Figure 12:**
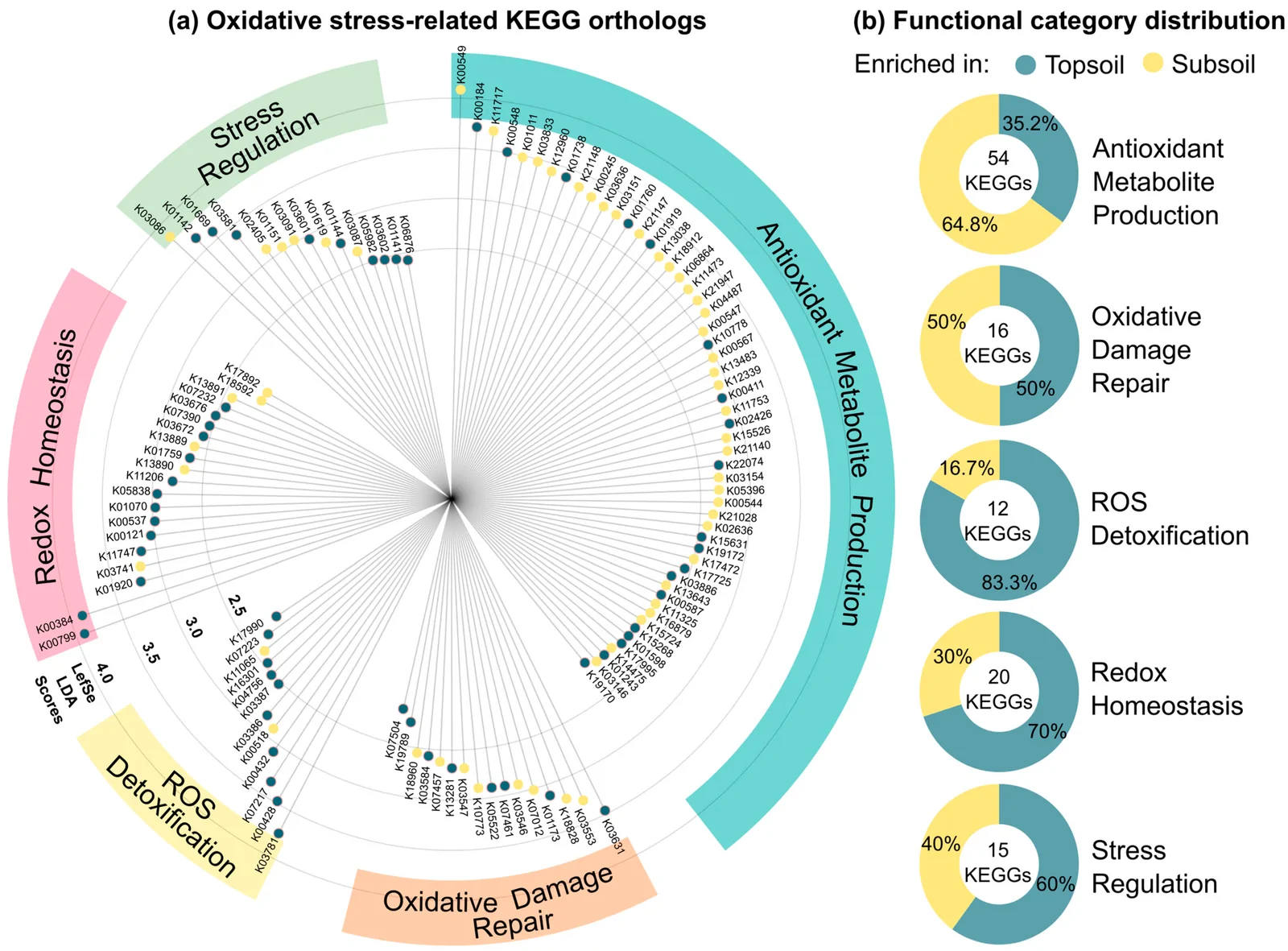
LEfSe biomarkers of oxidative stress-related KEGG orthologs across soil depths. (**a**) Circular visualization of the 117 significantly enriched oxidative stress-related KEGG orthologs identified by LEfSe analysis. Biomarkers are grouped into five manually curated oxidative stress-related functional categories: Antioxidant Metabolite Production, Oxidative Damage Repair, ROS Detoxification, Redox Homeostasis, and Stress Regulation. Each point represents a significant KEGG ortholog and is colored according to the soil compartment in which it was significantly enriched (Topsoil or Subsoil). Radial lines connect each biomarker to its corresponding LDA score, while the colored outer sectors indicate the functional category to which each KEGG ortholog belongs. (**b**) Donut charts summarizing the relative distribution of Topsoil- and Subsoil-enriched KEGG orthologs within each functional category. Percentages represent the proportion of significant biomarkers assigned to each soil compartment, and the value in the center of each donut indicates the total number of significant KEGG orthologs belonging to that category. The complete list of significant oxidative stress-related KEGG biomarkers identified by LEfSe is provided in Supplementary Materials (Table S2).

To identify the oxidative stress-related functions driving the functional differentiation between topsoil and subsoil microbial communities, LEfSe analysis was performed using oxidative stress-associated KEGG orthologs (Figure 12). A total of 117 significantly enriched KEGG biomarkers were identified, revealing clear depth-dependent functional specialization (Figure 12a). Topsoil was primarily characterized by biomarkers involved in redox balance, ROS detoxification, and cellular stress responses, including thioredoxin reductase (K00384), superoxide dismutase (K03781), and several DNA repair and stress-regulation functions. In contrast, subsoil communities were predominantly enriched in biomarkers associated with antioxidant metabolite production, including K00549, K01011, K03833, K12960, K21148, K00245, and K03636.

Grouping the significant biomarkers into five oxidative stress-related functional categories further emphasized these depth-dependent patterns (Figure 12b). Antioxidant Metabolite Production represented the largest functional group (*n* = 54), with 64.8% of biomarkers enriched in subsoil and 35.2% in topsoil. Oxidative Damage Repair contained 16 significant biomarkers and exhibited an equal distribution between topsoil and subsoil (50% each). In contrast, ROS Detoxification showed the strongest topsoil preference, with 83.3% of significant biomarkers enriched in topsoil and only 16.7% in subsoil. Likewise, Redox Homeostasis was predominantly represented by topsoil biomarkers (70%) compared with subsoil (30%), while Stress Regulation also favored topsoil (60%) over subsoil (40%).

#### 3.8.3. Nutrient Cycling and Metabolic Pathways

**Figure 13:**
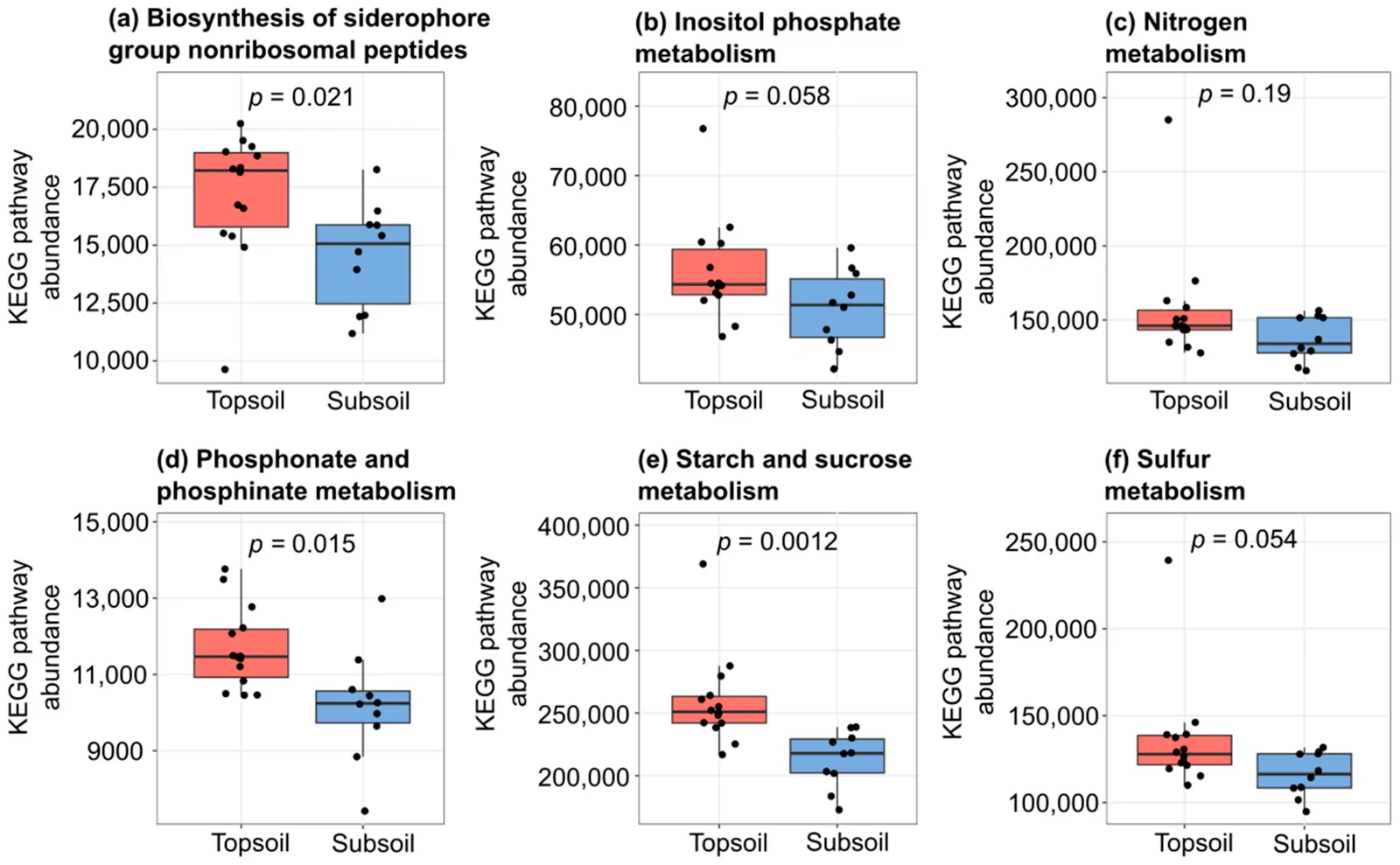
Depth-related variation in nutrient cycling and carbon metabolism pathways in crop soils (feed corn and sweet corn). Boxplots show the KEGG pathway abundance of selected metabolic pathways associated with biosynthesis of siderophore group nonribosomal peptides (**a**), inositol phosphate metabolism (**b**), nitrogen metabolism (**c**), phosphonate and phosphinate metabolism (**d**), starch and sucrose metabolism (**e**), and sulfur metabolism (**f**). Differences between topsoil and subsoil were evaluated using the Wilcoxon rank-sum test.

Selected KEGG pathways associated with carbon, nitrogen, phosphorus, sulfur, and iron metabolism were compared between topsoil and subsoil to investigate depth-related functional shifts (Figure 13). The results indicate that topsoil harbors a greater functional potential for several key metabolic processes. The biosynthesis of siderophore group nonribosomal peptides was significantly enriched in topsoil (*p* = 0.021). Similarly, phosphonate and phosphinate metabolism showed significantly higher KEGG pathway abundance in topsoil (*p* = 0.015). Among all pathways examined, starch and sucrose metabolism exhibited the strongest difference between soil layers (*p* = 0.0012). Inositol phosphate metabolism (*p* = 0.058) and sulfur metabolism (*p* = 0.054) displayed similar trends, with higher KEGG pathway abundance in topsoil, although the differences remained slightly above the conventional significance threshold. In contrast, nitrogen metabolism did not differ significantly between topsoil and subsoil (*p* = 0.19).

### 3.9. Communities of Species with Reported Phytopathogenic Potential

#### 3.9.1. Overall Prevalence of Species with Reported Phytopathogenic Potential Across Agricultural Systems

**Figure 14:**
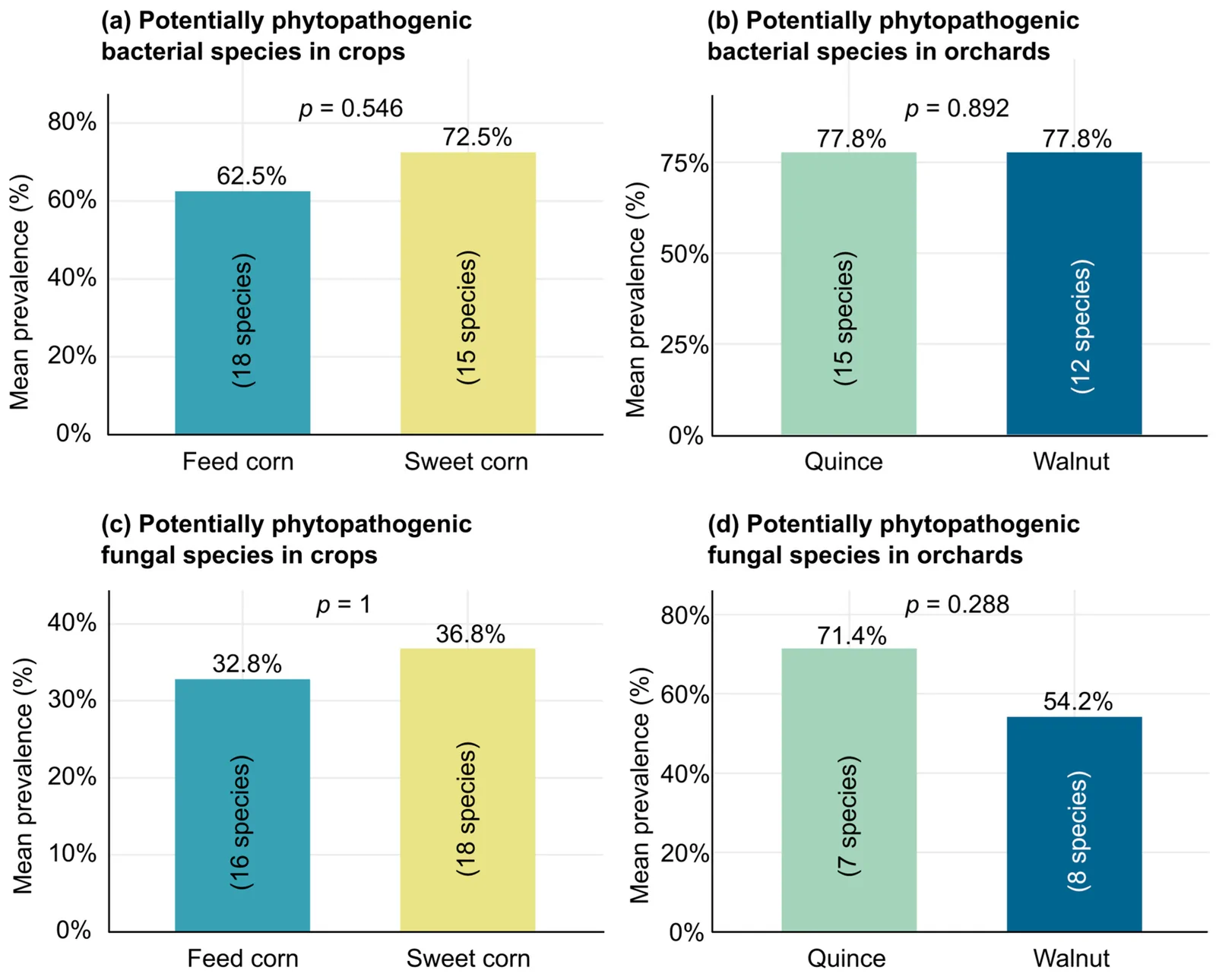
Normalized prevalence of bacterial and fungal species with reported phytopathogenic potential across crop and orchard systems. (**a**) Potentially phytopathogenic bacterial species in crops (feed corn and sweet corn), (**b**) potentially phytopathogenic bacterial species in orchards (quince and walnut), (**c**) potentially phytopathogenic fungal species in crops, and (**d**) potentially phytopathogenic fungal species in orchards. Bars represent the mean prevalence of detected pathogens, calculated as the average proportion of samples in which each pathogen was present within each group. Values inside bars indicate mean prevalence percentages and the number of detected pathogens. Prevalence was calculated only for species with reported phytopathogenic potential identified based on published evidence of phytopathogenicity and does not represent their proportion within the total soil microbiome. Statistical differences between groups were assessed using the Wilcoxon rank-sum test, with *p*-values and significance levels indicated above each panel. The complete pathogen lists are provided in the Supplementary Materials (Table S3).

To provide an initial overview of the occurrence of species with reported phytopathogenic potential across agricultural systems, their prevalence was examined separately for crop and orchard soils (Figure 14). Mean prevalence values were calculated only for the selected species with reported phytopathogenic potential and represent the average proportion of samples in which each species was detected within a given agricultural system. Therefore, these values describe the frequency of detection of these species across samples and do not represent their proportion within the total soil microbial community. Among crop systems, bacterial species with reported phytopathogenic potential exhibited relatively high prevalence in both feed corn and sweet corn soils. Sweet corn showed a higher mean bacterial pathogen prevalence (72.5%) than feed corn (62.5%), despite harboring a lower prevalence of detected bacterial species with reported phytopathogenic potential (15 vs. 18). However, the difference between crop types was not statistically significant (Wilcoxon test, *p* = 0.546).

A similar trend was observed for fungal species with reported phytopathogenic potential. Sweet corn displayed a slightly higher mean prevalence (36.8%) than feed corn (32.8%) and contained a greater number of detected fungal pathogen species (18 vs. 16). Nevertheless, prevalence distributions did not differ significantly between the two crop types (*p* = 1.000).

Within orchard systems, bacterial species with reported phytopathogenic potential were nearly identical between quince and walnut soils, with both systems exhibiting a mean prevalence of 77.8%. Although quince contained a larger number of detected bacterial pathogen species (15) than walnut (12), prevalence patterns remained highly similar (*p* = 0.892).

The largest contrast was observed for fungal phytopathogenic species in orchard soils. Quince exhibited a substantially higher mean fungal pathogen prevalence (71.4%) than walnut (54.2%), despite containing slightly fewer pathogen species (7 vs. 8).

### 3.9.2. Distribution of Potentially Phytopathogenic Species Across Agricultural Systems

**Figure 15:**
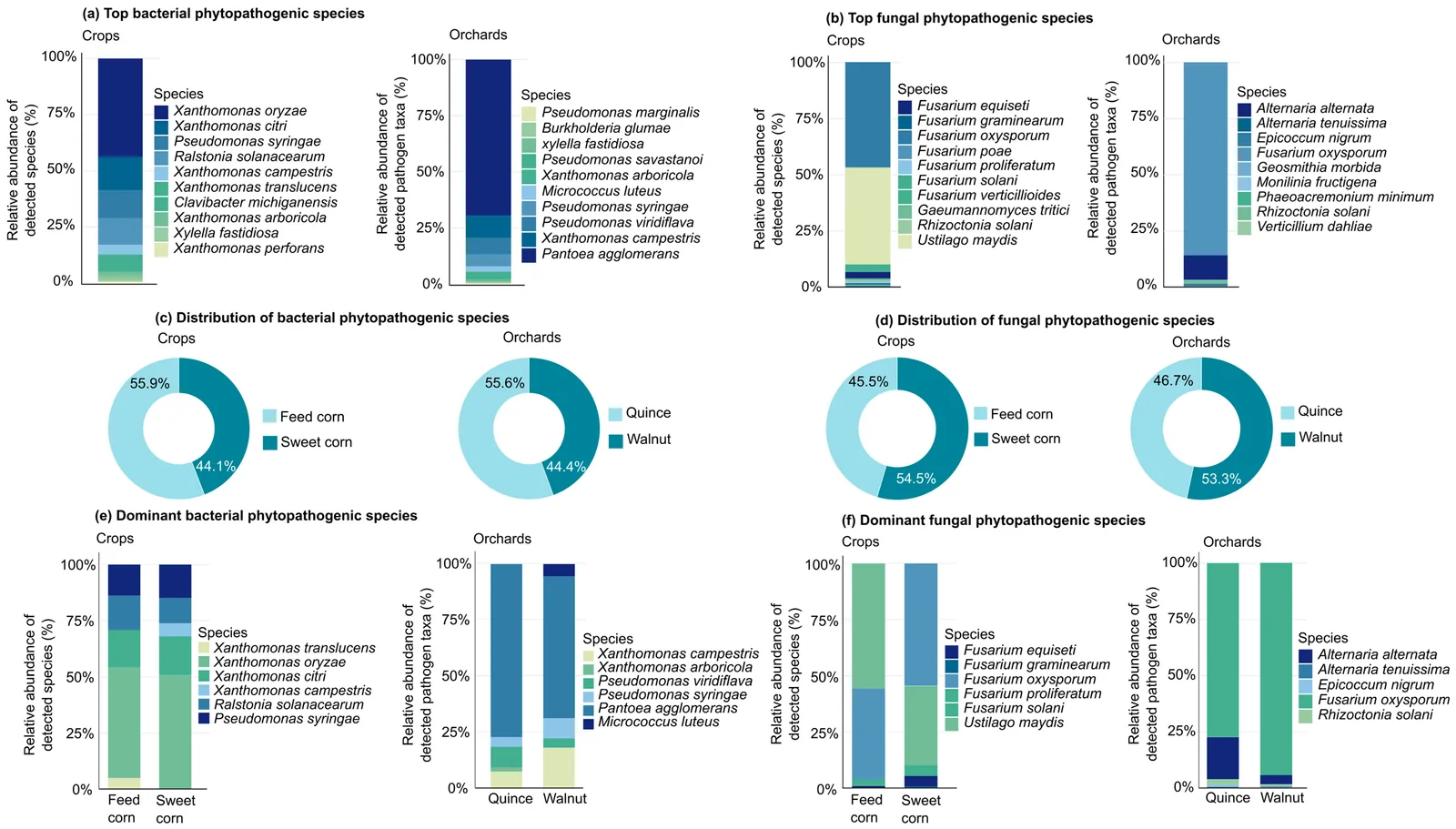
Integrated analysis of species with reported phytopathogenic potential across crop and orchard systems. Panels (**a**,**b**) show the relative abundance of the selected top bacterial and fungal species with reported phytopathogenic potential in Crops (feed corn and sweet corn) and Orchards (walnut and quince), respectively. Each stacked bar was normalized to 100% and illustrates the relative contribution of each species within the selected subset rather than within the total soil microbial community. Panels (**c**,**d**) present the distribution of bacterial and fungal species with reported phytopathogenic potential within crop and orchard systems, expressed as the percentage contribution of each crop type (feed corn vs. sweet corn) or orchard type (quince vs. walnut) to the total number of detected species. Panels (**e**,**f**) illustrate the relative abundance of the dominant bacterial and fungal species with reported phytopathogenic potential within each crop or orchard type, with each bar normalized to 100%, highlighting differences in species dominance among feed corn, sweet corn, quince, and walnut. Abundances were calculated only from the curated subset of detected species with reported phytopathogenic potential and do not represent their relative abundance within the total soil microbiome.

A substantial proportion of the curated list of species with reported phytopathogenic potential was detected within the studied soils, indicating that both crop and orchard systems harbored diverse communities of potentially phytopathogenic species (Figure 15). Potentially phytopathogenic bacterial assemblages in crop soils were dominated by members of the genera *Xanthomonas*, *Pseudomonas*, *Ralstonia*, *Clavibacter*, and *Xylella* (Figure 15a). In contrast, orchard soils were characterized primarily by *Pseudomonas marginalis*, *Burkholderia glumae*, *Xylella fastidiosa*, and several *Xanthomonas* species (Figure 14a). Potentially phytopathogenic fungal communities displayed even clearer differences between production systems (Figure 15b). Crop soils were dominated by *Fusarium*-associated pathogens, including *Fusarium oxysporum*, *F. graminearum*, *F. proliferatum*, *F. verticillioides*, and *F. solani*, whereas orchard soils were characterized by *Alternaria alternata*, *Epicoccum nigrum*, *Fusarium oxysporum*, *Monilinia fructigena*, and *Verticillium dahliae*.

Detected diversity of species with reported phytopathogenic potential was relatively evenly distributed among host types within each production system. Among bacterial species with reported phytopathogenic potential, feed corn contributed 55.9% of the crop-associated species, while sweet corn accounted for 44.1%. A similar pattern was observed in orchards, where quince and walnut contributed 55.6% and 44.4% of the detected bacterial phytopathogenic species diversity, respectively (Figure 15c). Fungal communities followed the same general trend, although sweet corn (54.5%) and walnut (53.3%) contributed slightly larger proportions of diversity of fungal species with reported phytopathogenic potential than feed corn (45.5%) and quince (46.7%) (Figure 15d).

Comparisons among individual host systems revealed additional differences in composition of species with reported phytopathogenic potential. Feed corn and sweet corn supported largely similar bacterial assemblages of species with reported phytopathogenic potential, although the relative contribution of individual *Xanthomonas* species varied between crops. Greater variation was observed within fungal communities, where sweet corn contained a larger contribution of *Fusarium* species than feed corn (Figure 15e). Orchard systems also differed in their species with reported phytopathogenic potential. Walnut soils showed a stronger dominance of *Monilinia fructigena*, whereas quince soils supported a more diverse fungal assemblage of species with reported phytopathogenic potential involving *Alternaria*, *Fusarium*, and *Rhizoctonia* species (Figure 15f). Similar host-specific shifts were observed among bacterial species with reported phytopathogenic potential, where the relative abundance of individual species varied between quince and walnut despite the presence of a broadly shared pathogen pool.

### 3.9.3. Species-Level Prevalence of Phytopathogenic Species

**Figure 16:**
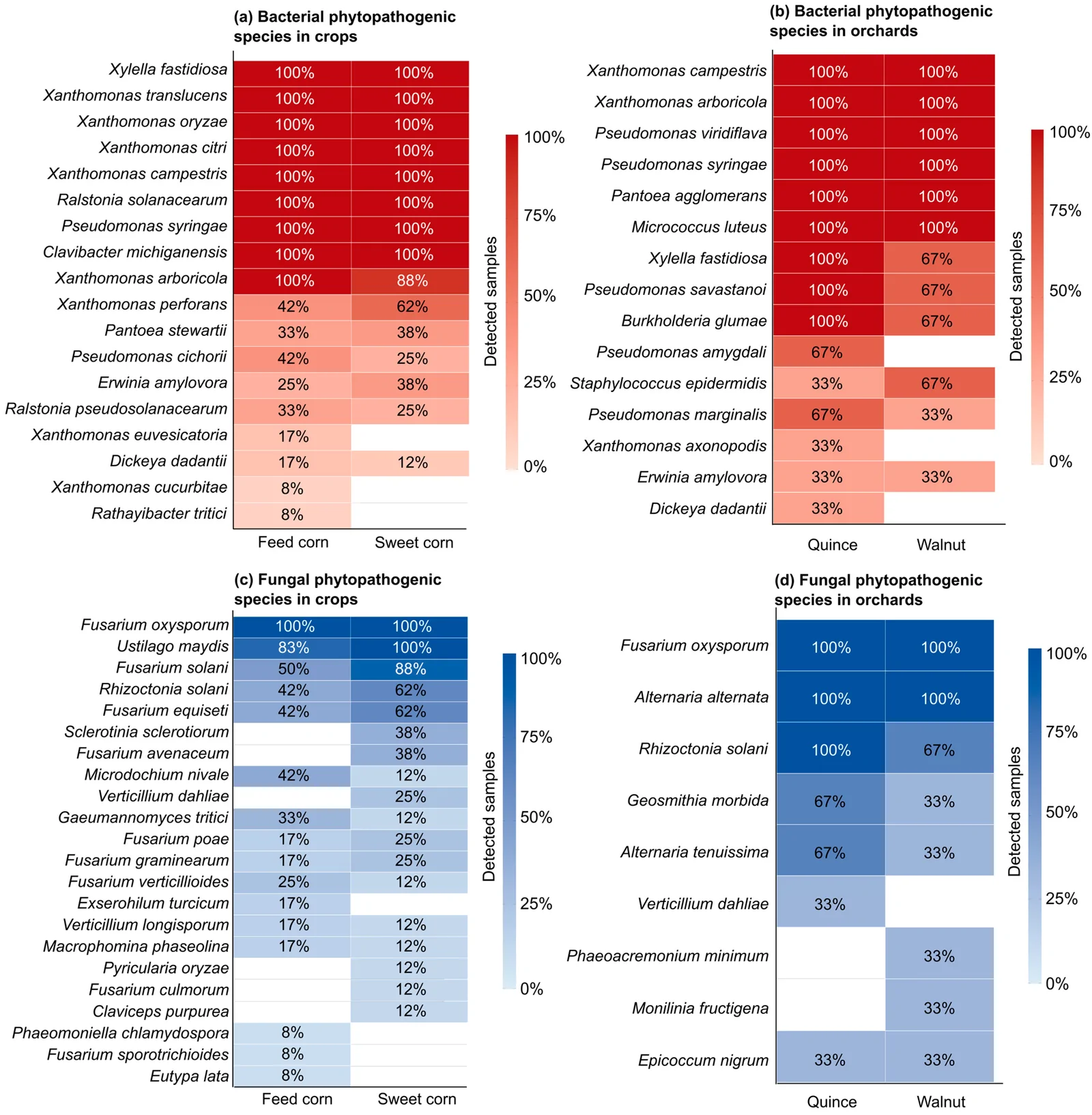
Prevalence patterns of bacterial and fungal species with reported phytopathogenic potential across crop and orchard systems based on species-level microbial profiling. Panels (**a**,**b**) show the prevalence of bacterial phytopathogenic species in crop (feed corn and sweet corn) and orchard (quince and walnut) systems, respectively, whereas panels (**c**,**d**) present the corresponding prevalence patterns of fungal phytopathogenic species. Prevalence was calculated as the proportion of samples within each crop type in which each species was detected and is expressed as a percentage. Only species detected in at least one sample were included in the analysis. Species are ordered according to their mean prevalence within each system. Color intensity reflects prevalence, with darker colors indicating more consistently detected species. Numerical values within cells represent prevalence percentages and indicate the frequency of species occurrence across samples rather than their relative abundance.

Species-level prevalence analyses revealed that a relatively small number of species with reported phytopathogenic potential occurred consistently across most samples, forming a core community of species with reported phytopathogenic potential within each production system (Figure 16). In contrast to abundance-based analyses, prevalence reflects the frequency with which individual species were detected and therefore identifies taxa that are widespread regardless of their relative abundance.

Crop-associated bacterial species with reported phytopathogenic potential exhibited particularly high prevalence (Figure 16a). *Xylella fastidiosa*, *Xanthomonas translucens*, *Xanthomonas oryzae*, *Xanthomonas citri*, *Xanthomonas campestris*, *Ralstonia solanacearum*, *Pseudomonas syringae*, and *Clavibacter michiganensis* were present in all feed corn and sweet corn samples, indicating a highly stable core of bacterial species with reported phytopathogenic potential. At the opposite end of the spectrum, *Dickeya dadantii*, *Xanthomonas cucurbitae*, and *Rathayibacter tritici* occurred only sporadically, with prevalence values ranging from 8% to 17%.

A similarly high prevalence was observed among orchard-associated bacterial species with reported phytopathogenic potential (Figure 16b). *Xanthomonas campestris*, *Xanthomonas arboricola*, *Pseudomonas viridiflava*, *Pseudomonas syringae*, *Pantoea agglomerans*, and *Micrococcus luteus* were detected in every quince and walnut sample. Other species, including *Xylella fastidiosa*, *Pseudomonas savastanoi*, and *Burkholderia glumae*, occurred in all quince samples but only two-thirds of walnut samples.

Among fungal species with reported phytopathogenic potential, *Fusarium oxysporum* was the most consistently detected species in crop systems, occurring in all feed corn and sweet corn samples (Figure 16c). *Ustilago maydis* also showed high prevalence, being present in 83% of feed corn and all sweet corn samples. Several additional *Fusarium* species, including *F. solani*, *F. equiseti*, and *F. proliferatum*, exhibited moderate to high prevalence, whereas *Eutypa lata*, *Fusarium sporotrichioides*, and *Phaeoacremonium chlamydospora* were encountered only occasionally.

Orchard fungal communities of fungal species with reported phytopathogenic potential displayed a somewhat different prevalence structure (Figure 16d). *Fusarium oxysporum* and *Alternaria alternata* were detected in all quince and walnut samples, making them the most consistently detected fungal species within orchard soils. *Rhizoctonia solani* was also highly prevalent, occurring in every quince sample and in 67% of walnut samples. In contrast, *Monilinia fructigena* and *Phaeoacremonium minimum* were restricted to walnut soils, whereas *Verticillium dahliae* was detected exclusively in quince soils.

### 3.9.4. Depth-Related Distribution of Phytopathogenic Species

**Figure 17:**
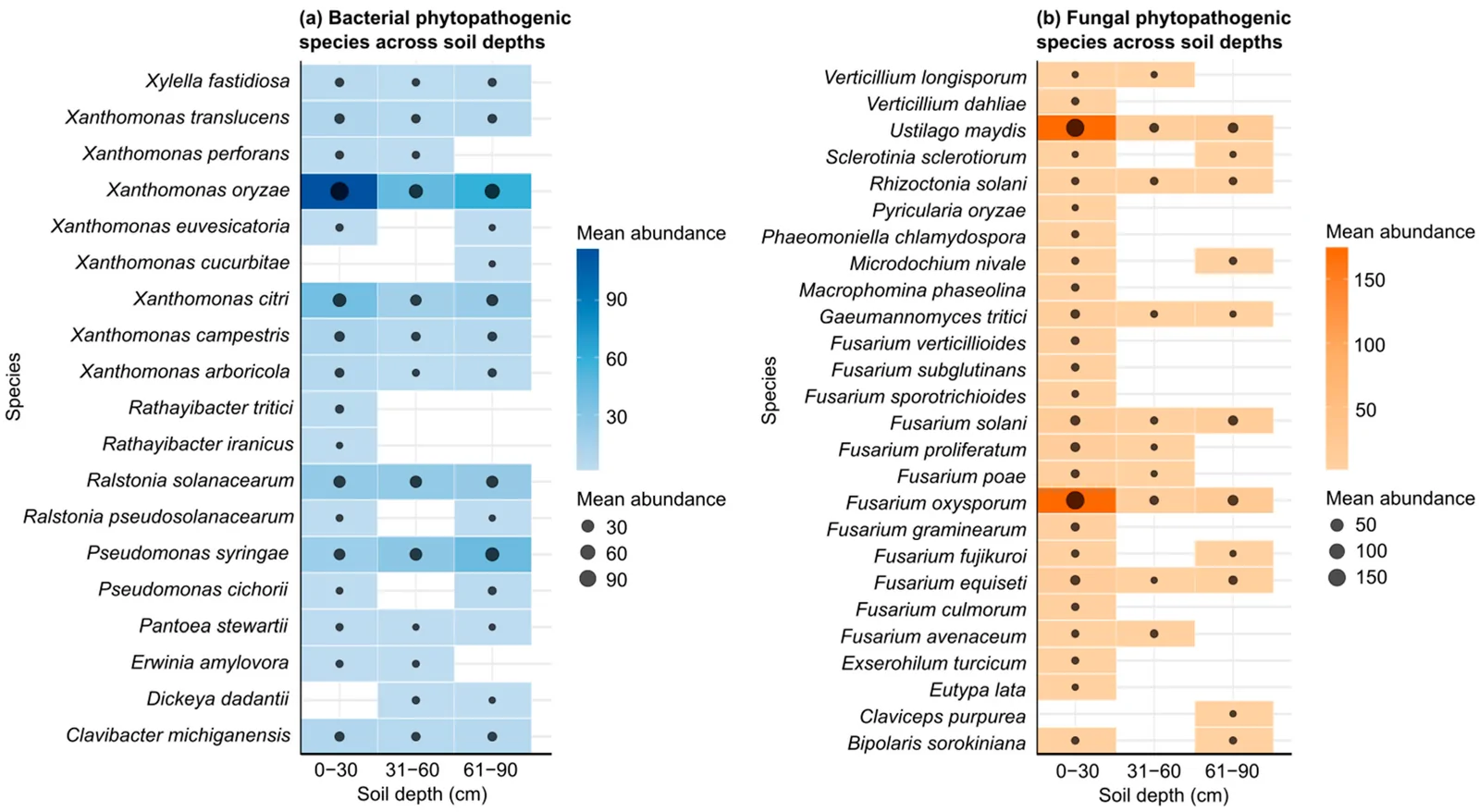
Depth-related distribution and mean abundance of crop-associated phytopathogenic species in agricultural soils. Panels show (**a**) bacterial and (**b**) fungal phytopathogenic species detected in crop systems (feed corn and sweet corn) across three soil depth intervals (0–30, 31–60, and 61–90 cm). Heatmap color intensity and bubble size both represent the mean abundance of each species calculated across all samples within each depth layer. Only species with reported phytopathogenic potential included in the curated crop-specific list and detected in the dataset are shown.

The widespread occurrence of several bacterial and fungal pathogens prompted a further examination of their vertical distribution within the soil profile. By comparing pathogen abundance across the 0–30, 31–60, and 61–90 cm soil layers, it was possible to assess whether phytopathogenic species were concentrated in surface soils or persisted throughout deeper horizons (Figure 17).

Bacterial phytopathogenic species displayed relatively broad depth distributions, with several taxa occurring across all three soil layers (Figure 17a). *Xanthomonas oryzae*, *Pseudomonas syringae*, *Ralstonia solanacearum*, *Xanthomonas citri*, and *Clavibacter michiganensis* maintained measurable abundance throughout the soil profile. Among these taxa, *Xanthomonas oryzae* exhibited the highest abundance, particularly within the 0–30 cm layer. In contrast, *Ralstonia pseudosolanacearum*, *Pseudomonas cichorii*, and *Xanthomonas cucurbitae* showed more restricted distributions and occurred only within selected depth intervals at comparatively low abundance.

Fungal phytopathogenic species exhibited stronger depth-related patterns than bacterial phytopathogenic species (Figure 17b). The highest abundance values were generally concentrated in the topsoil, where *Fusarium oxysporum* and *Ustilago maydis* emerged as the dominant fungal phytopathogenic species. Several *Fusarium* species, including *F. solani*, *F. proliferatum*, *F. poae*, and *F. equiseti*, were detected across multiple soil layers, demonstrating their ability to persist throughout the soil profile. Other fungal phytopathogenic species displayed more distinct depth preferences. *Verticillium longisporum*, *Phaeomoniella chlamydospora*, and *Macrophomina phaseolina* were primarily associated with the upper soil layer, whereas *Claviceps purpurea* and *Bipolaris sorokiniana* were more frequently detected in deeper horizons.

### 3.9.5. Environmental Drivers of Phytopathogenic Species Distribution

**Figure 18:**
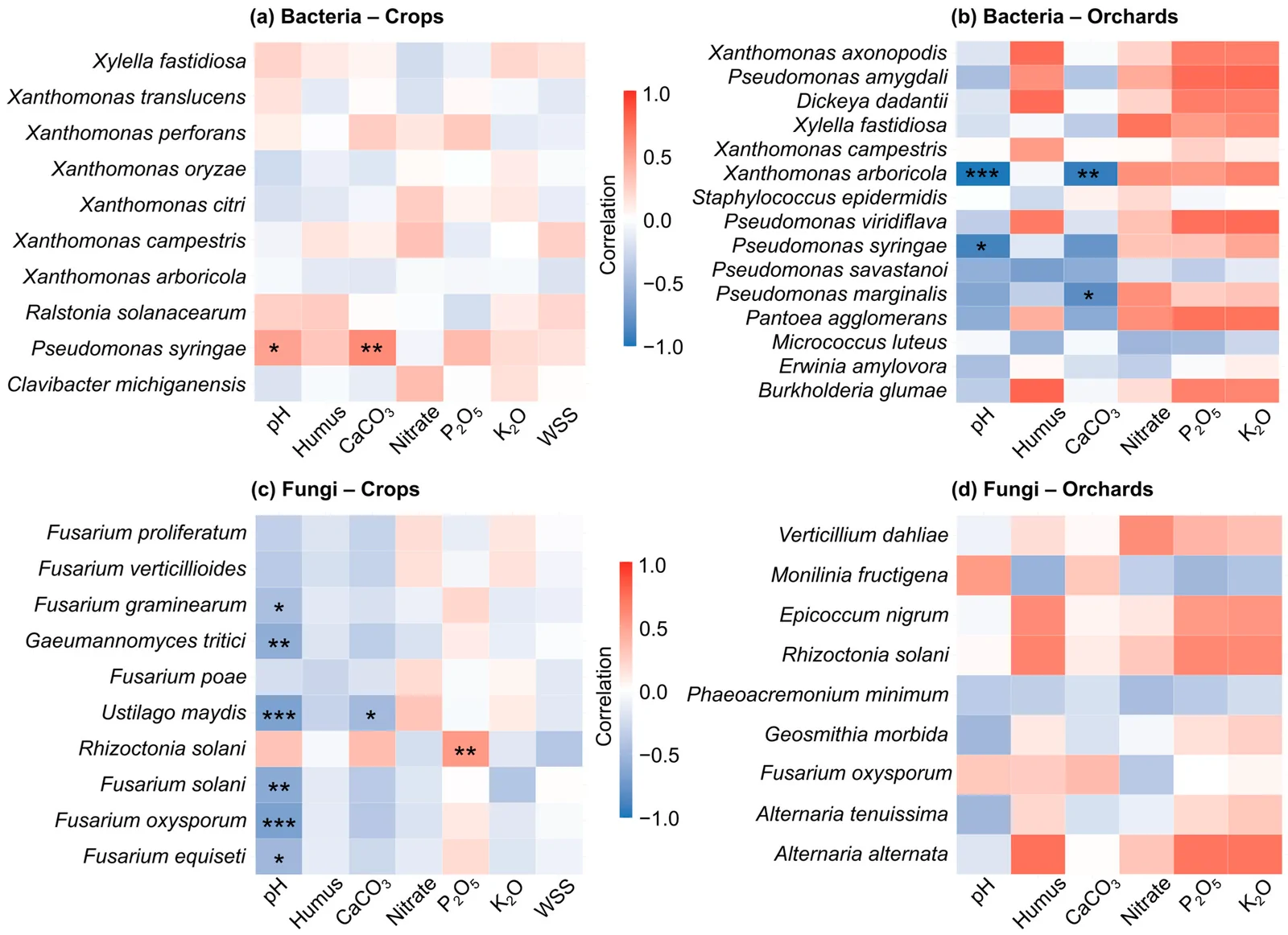
Relationships between soil physicochemical properties and the most abundant plant species with reported phytopathogenic potential in crop and orchard systems. Panels (**a**,**b**) show Pearson correlation coefficients between the abundance of bacterial phytopathogenic species and soil properties in crops and orchards, respectively, while panels (**c**,**d**) present the corresponding analyses for fungal phytopathogenic species. Heatmap colors represent the direction and strength of correlations, ranging from negative (blue) to positive (orange) relationships. Asterisks indicate statistically significant correlations (* *p* < 0.05, ** *p* < 0.01, *** *p* < 0.001). Only the ten most abundant species within each system were included. Soil variables comprise pH, humus content, CaCO_3_, nitrate, available P_2_O_5_, available K_2_O, and WSS where available.

The widespread occurrence of several phytopathogenic species across crop and orchard systems prompted an examination of their relationships with soil physicochemical properties (Figure 18). Overall, crop-associated fungal phytopathogenic species exhibited the strongest and most frequent associations with soil variables, whereas phytopathogenic species in orchard soils generally showed weaker and predominantly non-significant responses.

Crop-associated bacterial phytopathogenic species generally exhibited weaker responses than fungal pathogens (Figure 18a). Most correlations were low to moderate and non-significant. The clearest associations were observed for *Pseudomonas syringae*, which showed positive correlations with pH (r ≈ 0.49, *p* < 0.05) and CaCO_3_ (r ≈ 0.61, *p* < 0.01). Other bacterial phytopathogenic species displayed only limited relationships with the measured soil variables.

A similar pattern was observed within orchard-associated bacterial species (Figure 18b). Most associations were weak and non-significant, although *Xanthomonas arboricola* exhibited a strong negative correlation with pH (r ≈ −0.81, *p* < 0.001) and a negative association with CaCO_3_ (r ≈ −0.69, *p* < 0.01). *Pseudomonas syringae* also showed a weaker negative relationship with pH (*p* < 0.05). Positive associations with nutrient-related variables such as nitrate, P_2_O_5_, and K_2_O were observed for several taxa, although these relationships were not statistically significant.

Among crop-associated fungal phytopathogenic species (Figure 18c), soil pH emerged as the most influential environmental factor. Several *Fusarium* species exhibited significant negative correlations with pH, including *Fusarium oxysporum* (r = −0.68, *p* < 0.001), *Fusarium solani* (r = −0.61, *p* < 0.01), *Fusarium equiseti* (r = −0.51, *p* < 0.05), and *Fusarium graminearum* (r = −0.45, *p* < 0.05). Similar negative relationships were observed for *Ustilago maydis* (r = −0.67, *p* < 0.001) and *Gaeumannomyces tritici* (r = −0.58, *p* < 0.01). In contrast, *Rhizoctonia solani* was positively associated with available phosphorus (P_2_O_5_) (r = 0.53, *p* < 0.01), while *U. maydis* also exhibited a negative relationship with CaCO_3_ content (r = −0.49, *p* < 0.05).

Relationships between orchard-associated fungal phytopathogenic species and soil properties were considerably weaker (Figure 18d). Although several taxa displayed moderate positive or negative trends, none of the observed correlations reached statistical significance.

## 4. Discussion

The results of this study demonstrate that soil microbial communities are shaped primarily by vertical soil stratification, whereas the effects of land-use depend on the microbial group considered. Soil depth consistently influenced both bacterial and fungal communities, while land-use and plant system exerted stronger effects on fungal than on bacterial community composition. Among all evaluated variables, soil depth emerged as the strongest determinant of microbial community composition, highlighting the importance of vertical environmental gradients in structuring belowground biodiversity.

The strong influence of depth is consistent with the concept of environmental filtering, whereby soil horizons represent distinct ecological habitats characterized by differences in carbon availability, root density, oxygen concentration, moisture dynamics, and nutrient accessibility. Surface soils receive continuous inputs of plant residues and root exudates that create diverse ecological niches and support highly heterogeneous microbial communities. In contrast, deeper soil layers are generally characterized by lower carbon availability and more stable environmental conditions, favoring a smaller subset of microorganisms adapted to resource limitation [75]. Consequently, the observed decline in richness with depth likely reflects not only reduced resource availability but also increasing environmental specialization. The pronounced separation of microbial communities among soil layers further suggests that depth-related patterns are driven primarily by species replacement rather than simple richness loss, indicating that deeper horizons harbor distinct microbial assemblages adapted to specific ecological conditions [76]. The correlation analyses further indicated that bacterial alpha diversity was only weakly associated with the measured soil physicochemical properties, whereas fungal diversity showed significant negative relationships with soil pH and CaCO_3_. These findings indicate that fungal communities were more responsive than bacterial communities to variation in soil chemical properties across the studied land-use systems. Because the investigated soils represent managed agricultural systems rather than undisturbed natural ecosystems, recurrent cultivation and other management practices may have partially reduced vertical stratification compared with natural soils. Therefore, the observed depth-related patterns should be interpreted within the context of managed agricultural ecosystems.

While depth represented the dominant environmental filter for both bacterial and fungal communities, the influence of land-use differed between the two microbial groups. Land-use significantly affected fungal community composition but had no detectable effect on bacterial community composition. Nevertheless, microbial richness remained largely unchanged across land-use systems, suggesting that land-use primarily altered community composition through species turnover rather than changes in overall richness. Such a pattern is frequently observed in soil ecosystems and reflects the high degree of functional redundancy within microbial communities. Multiple taxa often perform similar ecological functions, allowing species turnover to occur without major changes in overall diversity or ecosystem functioning [77]. Similarly, plant system influenced fungal but not bacterial community composition, indicating that orchard and crop soils supported compositionally distinct fungal assemblages despite exhibiting comparable levels of alpha diversity. The stronger response of fungal communities to land-use and plant system observed in the present study agrees with previous findings demonstrating that fungal community structure in agricultural soils is strongly influenced by agricultural management and host-specific plant interactions, with tillage practices and crop identity acting as important selective forces [78].

At the same time, the identification of numerous unique bacterial and fungal taxa indicates that individual land-use systems provide distinct ecological niches that support specialized microbial assemblages [79]. The particularly high number of unique taxa detected in crop soils may reflect the greater environmental heterogeneity associated with annual cultivation, fertilization practices, seasonal vegetation turnover, and recurrent soil disturbance [79]. Although the present study did not directly compare different agricultural management practices, the observed microbial patterns should be interpreted within the broader context of agricultural soil management. Practices such as ploughing, rotary cultivation, subsoiling, fertilization, and crop rotation are known to modify soil structure, resource distribution, and habitat heterogeneity, thereby influencing the vertical organization of soil microbial communities. Consequently, the depth- and land-use-related patterns observed here likely reflect not only natural environmental gradients but also the cumulative effects of long-term agricultural management. Furthermore, the managed non-cropped agricultural reference site was also subjected to occasional shallow soil loosening and ploughing for land maintenance. Although these interventions were substantially less intensive than those applied in cropped systems, they may have influenced soil structure, aeration, organic matter distribution, and microbial community composition. Therefore, the observed differences should be interpreted as reflecting contrasting agricultural management regimes, particularly cropped versus managed non-cropped soils, rather than cultivation effects relative to a completely undisturbed ecosystem. Future studies explicitly comparing contrasting management systems would help clarify the relative contribution of individual management practices to microbial stratification, ecological functioning, and soil health.

Fungal communities appeared to respond more strongly to land-use differences than bacterial communities, a pattern commonly reported in agricultural ecosystems. Unlike bacteria, fungi rely heavily on plant-derived carbon inputs and frequently establish close associations with roots, litter, and decomposing plant residues [80]. As a result, differences among alfalfa, crops, orchards, and uncultivated systems are likely to create contrasting resource environments that selectively favor distinct fungal assemblages. Bacterial communities, although responsive to environmental change, generally exhibit greater metabolic flexibility and are therefore capable of persisting across a wider range of environmental conditions [81]. This difference in ecological strategy may explain why fungal communities exhibited stronger compositional turnover than bacterial communities across the studied land-use systems.

The observed functional differentiation indicates that the effects of land-use extend beyond taxonomic composition and ultimately influence ecological processes linked to nutrient cycling, plant productivity, and carbon turnover [82,83]. However, the strength of this response varied among functional groups. Functions associated with phosphorus and potassium solubilization, plant growth promotion, mycorrhizal symbiosis, hydrocarbon degradation, and organic matter decomposition exhibited the clearest land-use-specific differentiation, whereas nitrogen fixation, bioremediation, and antibiotic resistance remained comparatively conserved after correction for multiple comparisons. These findings suggest that microbial functions directly linked to nutrient acquisition and carbon turnover are more responsive to long-term changes in vegetation and management practices than broadly distributed ecological functions [84]. The particularly strong differentiation observed for organic matter decomposition, which exhibited the highest number of significant pairwise comparisons among all evaluated functional groups, further highlights the importance of organic matter quality in shaping microbial communities. Although microbial communities exhibit a considerable degree of functional redundancy, certain ecosystem processes remain strongly dependent on community composition. Glassman et al. [85] demonstrated that shifts in microbial composition can directly alter decomposition rates and litter chemistry, suggesting that different vegetation types support microbiomes with distinct functional capacities for degrading cellulose, lignin, and other organic compounds. Therefore, the functional divergence observed among land-use systems likely reflects differences in both resource quality and long-term vegetation inputs.

Despite substantial taxonomic and functional turnover, microbial dominance patterns remained remarkably stable across land-use systems. In all cases, a relatively small fraction of taxa accounted for the majority of microbial abundance, whereas most species occurred at low abundance. This pattern is characteristic of complex microbial ecosystems and reflects the existence of a stable abundance hierarchy. Dominant taxa are often ecological generalists capable of efficiently exploiting available resources, while rare taxa constitute a large reservoir of genetic and functional diversity that contributes to ecosystem resilience. Similar patterns were reported by Gschwend et al. [86], who showed that the relationship between abundant and rare taxa remains surprisingly consistent across contrasting land-use systems despite ongoing species turnover.

Although dominance structure remained largely unchanged, network analyses revealed pronounced differences in the organization of microbial interactions. This apparent contrast suggests that land-use systems influence how microorganisms interact rather than how abundance is distributed among taxa. The highly connected bacterial and fungal networks observed in alfalfa soils likely result from the continuous root inputs and reduced disturbance associated with perennial vegetation. Long-term carbon inputs can promote cooperative interactions and increase network stability, leading to more integrated microbial communities. Conversely, the reduced connectivity observed in crop soils may reflect periodic disturbance, seasonal vegetation turnover, and fluctuating resource availability [87].

Depth-related network responses revealed an equally interesting pattern. Although deeper soil layers contained fewer species, the remaining microorganisms formed increasingly interconnected networks. One possible explanation is that resource limitation strengthens microbial dependencies, promoting cooperative interactions, cross-feeding relationships, and metabolic complementarity among the taxa that persist in deeper horizons. Similar mechanisms have been proposed for oligotrophic environments, where community stability relies more heavily on interspecific interactions than on taxonomic diversity itself. However, the direction of depth-related network responses is not universal. Guseva et al. [88] reported lower connectivity in forest subsoils, suggesting that the effects of depth depend strongly on ecosystem type, vegetation cover, and local environmental conditions.

Soil depth strongly influenced the oxidative stress response strategies of microbial communities. The enrichment of ROS detoxification, redox homeostasis, and stress-regulation functions in topsoil suggests that microorganisms in surface horizons are exposed to greater oxidative stress due to higher oxygen availability, stronger environmental fluctuations, and intensive root activity. According to Pett-Ridge and Firestone [89], microbes in the surface layers of tropical soils, exposed to strong environmental fluctuations, develop physiological tolerance mechanisms and antioxidant enzymes to cope with redox stress. The higher representation of ROS-detoxification functions in topsoil suggests a greater genomic potential of microbial communities to withstand oxidative stress under fluctuating environmental conditions. This potential may help maintain microbial processes involved in organic matter decomposition and nutrient mineralization, thereby supporting nutrient availability and soil fertility and, indirectly, plant performance [90]. Similarly, the enrichment of functions related to redox homeostasis may reflect an enhanced capacity to maintain intracellular redox balance, preserve enzyme activity, and sustain microbial metabolism during changes in moisture, temperature, oxygen availability, and root-derived substrate inputs. The greater representation of stress-regulation functions is also consistent with increased microbial resilience, potentially enabling topsoil communities to adapt rapidly to environmental perturbations while maintaining key biogeochemical processes [91,92]. In contrast, the enrichment of functions associated with antioxidant metabolite biosynthesis in subsoil may represent a strategy supporting microbial persistence under relatively stable but resource-limited conditions. These metabolites may protect microbial cells from cumulative oxidative damage and contribute to the maintenance of microbial activity in deeper soil horizons. Their potential influence on soil carbon persistence is likely indirect and may depend on interactions among microbial activity, substrate availability, and the turnover of organic matter [93,94]. The enrichment of thioredoxin reductase- and superoxide dismutase-related functions further supports the importance of active oxidative stress management in topsoil microbial communities. Although oxidative damage-repair functions did not differ significantly in their overall representation between soil layers, their consistent occurrence throughout the soil profile highlights their fundamental role in maintaining DNA and protein integrity under both dynamic surface conditions and the comparatively stable environment of deeper soil horizons. These KEGG-based patterns should nevertheless be interpreted as indicators of functional potential rather than direct evidence of metabolic activity.

Depth also affected nutrient-related metabolic pathways. The higher KEGG pathway abundance of siderophore biosynthesis, phosphorus-acquisition pathways, and carbohydrate metabolism in topsoil likely reflects greater microbial competition for nutrients and increased availability of plant-derived substrates. According to Huang et al. [95], soil depth significantly influences the composition of soil organic matter, with topsoil characterized by a higher abundance of carbohydrates and a greater availability of plant-derived substrates, such as plant residues and root exudates, compared with subsoil. These findings suggest that soil depth selects for distinct microbial functional strategies, with topsoil communities adapted for rapid stress response and resource acquisition, whereas subsoil communities appear better adapted to relatively stable and resource-limited conditions.

Communities of species with reported phytopathogenic potential provided an additional perspective on how environmental conditions shape microbial assemblages. The widespread occurrence of several bacterial and fungal pathogens suggests the existence of stable cores of phytopathogenic species within both crop and orchard systems. However, prevalence alone should not be interpreted as disease risk because pathogen activity depends on interactions among host plants, environmental conditions, and surrounding microbial communities. The detection of species with reported phytopathogenic potential in soil should not be equated with active pathogenicity or disease risk. Although shotgun metagenomic sequencing provides valuable information on the composition and prevalence of pathogen-associated genomic signatures, it does not determine whether the detected organisms are viable, metabolically active, virulent, or capable of infecting host plants. Moreover, detected DNA may originate from inactive or non-viable cells. Disease establishment requires the convergence of a susceptible host, a viable and virulent pathogen, and conducive environmental conditions. Therefore, the prevalence patterns reported in this study should be regarded as indicators of the potential distribution of species with reported phytopathogenic potential in agricultural soils rather than as direct evidence of disease occurrence. Host infection assays, viability-based approaches, or functional analyses would be required to confirm pathogenic activity.

Among the evaluated soil properties, pH emerged as the most consistent predictor of the abundance of phytopathogenic species. The strong negative associations observed between pH and several *Fusarium* species support the view that soil acidity acts as a major ecological filter regulating the persistence of phytopathogenic species and competitiveness. This interpretation is consistent with the findings of Li et al. [96], who identified soil pH as one of the strongest predictors of *Fusarium* root rot severity and demonstrated that soil acidification reduces the natural suppressive capacity of the soil microbiome. In contrast, nutrient-related variables exhibited comparatively weak relationships with abundance of phytopathogenic species, indicating that distribution of phytopathogenic species is influenced more strongly by environmental filtering and biological interactions than by nutrient availability alone.

The weaker environmental responses observed among orchard-associated phytopathogenic species may reflect the greater ecological stability of perennial systems. Compared with annually cultivated fields, orchard soils experience lower levels of physical disturbance and maintain long-term plant–microbe associations that can buffer short-term fluctuations in environmental conditions. Similar patterns have been reported in perennial fruit systems, where management history and landscape context often exert stronger influences on microbial community assembly than individual soil properties [97]. Together, these findings suggest that communities of phytopathogenic species are governed by a combination of abiotic filtering, host specificity, and microbial interactions, with soil pH representing one of the most important environmental drivers identified in the present study.

Collectively, these findings indicate that soil depth acts as the primary environmental filter shaping microbial community assembly, whereas land-use systems primarily influence community composition, ecological functions, and microbial interactions. Although richness and dominance structure remained relatively stable, substantial turnover was observed in taxonomic composition, functional potential, and network organization. The stronger response of fungal communities compared with bacterial communities highlights the importance of vegetation-driven resource availability in structuring soil microbiomes. Together, these results demonstrate that both vertical stratification and land-use history contribute to the development of functionally distinct soil microbial communities with potential implications for nutrient cycling, ecosystem stability, and plant health.

## 5. Conclusions

Understanding how agricultural management and environmental gradients shape soil microbial communities is essential for maintaining soil health and ecosystem sustainability. The results highlight the importance of soil depth and land-use system in shaping agricultural soil microbiomes. Soil depth emerged as the dominant factor influencing microbial richness, community composition, and network structure in both bacterial and fungal communities, whereas land-use effects were more pronounced for fungal than bacterial community composition and primarily influenced taxonomic turnover, ecological functions, microbial interactions, and the distribution of phytopathogenic species. Despite relatively stable alpha diversity across most land-use systems, substantial differences were observed in community composition, functional potential, and network organization. Fungal communities responded more strongly than bacterial communities to land-use system and plant system differences, whereas soil depth consistently structured both microbial groups. In addition, soil depth was associated with distinct oxidative stress response strategies, with topsoil communities exhibiting greater potential for ROS detoxification, redox homeostasis, and stress regulation, while subsoil communities were characterized by a higher representation of antioxidant metabolite production functions. Network analyses further revealed greater connectivity in perennial systems, particularly alfalfa soils, while analyses of phytopathogenic species identified soil pH as one of the most important factors associated with their distribution. Overall, these findings demonstrate that agricultural management influences not only microbial diversity but also microbial community composition, ecological functions, and interactions that underpin soil health and ecosystem functioning. Future studies should combine multi-omics approaches with broader geographical sampling to better understand the mechanisms governing soil microbiome assembly and resilience under different management practices. Such approaches may also help elucidate how beneficial soil microbiomes influence crop nutritional quality and the accumulation of health-promoting bioactive compounds, thereby supporting the development of sustainable agricultural systems for future medical food production.

## 6. Limitations

A limitation of the present study is that sampling was conducted only once during the autumn season. Consequently, the observed microbial patterns represent a temporal snapshot and do not capture seasonal dynamics or microbial responses to soil disturbance. Future studies incorporating repeated sampling across seasons or before and after management interventions would provide a more comprehensive understanding of microbial resilience, recovery, and functional changes under agricultural management. In addition, because all land-use systems were located within the same experimental area, spatial proximity may have reduced differences among them. Also, detailed historical information on previous management practices was not available for all land-use systems, which may have influenced the observed microbial community patterns. Future studies including spatially independent sites would help to confirm the generality of these findings. Finally, a limitation of the literature-based functional assignment is that microbial taxa may perform multiple ecological functions depending on environmental conditions, whereas published evidence is often derived from specific experimental settings. Consequently, the assigned functional groups represent the reported ecological potential of the detected taxa rather than direct evidence of their functional activity in the investigated soils.

## Data Availability

The original metagenomic sequencing data generated in this study are publicly available in the NCBI Sequence Read Archive (SRA) under BioProject accession number PRJNA1495987.

## Supplementary Materials

The following supporting information can be downloaded at: https://www.mdpi.com/article/10.3390/antiox15081017/s1. Supplementary Table S1. Literature-based classification of oxidative stress-related functional categories used for metagenomic analyses. Supplementary Table S2. Complete LEfSe results for oxidative stress-related KEGG orthologs differentiating topsoil and subsoil microbial communities. Supplementary Table S3. Bacterial and fungal species with reported phytopathogenic potential included in crop and orchard systems.
