# Supplementary material for: Land-Use and Depth-Dependent Assembly of Soil Microbiomes Shapes Ecological Functions, Interaction Networks, and Phytopathogenic Communities Across Crop and Orchard Systems
Source: Antioxidants (Basel). 2026 Aug 14;15(8):1017. doi: 10.3390/antiox15081017 (PMC13509613; doi:10.3390/antiox15081017)
Supplement: Supplementary file 1 [file antioxidants-15-01017-s001.zip › antioxidants-4440162-supplementary S1_oxidative_groups.pdf]

Supplementary Table S1. Literature-based classification of oxidative stress-related functional categories used for metagenomic analyses.

Group	Includes	Importance	References
**ROS Detoxification**	Superoxide dismutase, catalase, peroxidases, peroxiredoxins, alkyl hydroperoxide reductases	Direct elimination of reactive oxygen species and prevention of oxidative damage.	[17-20]
**Redox Homeostasis Systems**	Glutathione system + Thioredoxin system + NADPH-related regeneration pathways	Maintains intracellular redox balance and regenerates antioxidant capacity.	[21-25]
**Oxidative Damage Repair**	DNA repair, protein repair, oxidative damage response pathways	Repairs cellular damage caused by ROS and ensures survival under oxidative stress.	[26-32]
**Oxidative Stress Sensing and Regulation**	OxyR, SoxR, SoxS and other stress-response regulators	Detects oxidative stress and activates protective antioxidant mechanisms.	[33-37]
**Antioxidant Metabolite Production**	Carotenoids, riboflavin-related pathways, sulfur/cysteine metabolism supporting antioxidant synthesis	Produces antioxidant molecules and precursors that protect cells against ROS.	[38-43]
